# Mechanisms and pathologies of human mitochondrial DNA replication and deletion formation

**DOI:** 10.1042/BCJ20230262

**Published:** 2024-05-28

**Authors:** Tiago M. Bernardino Gomes, Amy E. Vincent, Katja E. Menger, James B. Stewart, Thomas J. Nicholls

**Affiliations:** 1 Translational and Clinical Research Institute, Faculty of Medical Sciences, Newcastle University, Newcastle upon Tyne NE2 4HH, U.K.; 2 Wellcome Centre for Mitochondrial Research, Faculty of Medical Sciences, Newcastle University, Newcastle upon Tyne NE2 4HH, U.K.; 3 NHS England Highly Specialised Service for Rare Mitochondrial Disorders, Newcastle upon Tyne Hospitals NHS Foundation Trust, Newcastle upon Tyne NE2 4HH, U.K.; 4 Biosciences Institute, Faculty of Medical Sciences, Newcastle University, Newcastle upon Tyne NE2 4HH, U.K.

**Keywords:** DNA damage, DNA replication and recombination, mitochondrial dysfunction, mtDNA

## Abstract

Human mitochondria possess a multi-copy circular genome, mitochondrial DNA (mtDNA), that is essential for cellular energy metabolism. The number of copies of mtDNA per cell, and their integrity, are maintained by nuclear-encoded mtDNA replication and repair machineries. Aberrant mtDNA replication and mtDNA breakage are believed to cause deletions within mtDNA. The genomic location and breakpoint sequences of these deletions show similar patterns across various inherited and acquired diseases, and are also observed during normal ageing, suggesting a common mechanism of deletion formation. However, an ongoing debate over the mechanism by which mtDNA replicates has made it difficult to develop clear and testable models for how mtDNA rearrangements arise and propagate at a molecular and cellular level. These deletions may impair energy metabolism if present in a high proportion of the mtDNA copies within the cell, and can be seen in primary mitochondrial diseases, either in sporadic cases or caused by autosomal variants in nuclear-encoded mtDNA maintenance genes. These mitochondrial diseases have diverse genetic causes and multiple modes of inheritance, and show notoriously broad clinical heterogeneity with complex tissue specificities, which further makes establishing genotype-phenotype relationships challenging. In this review, we aim to cover our current understanding of how the human mitochondrial genome is replicated, the mechanisms by which mtDNA replication and repair can lead to mtDNA instability in the form of large-scale rearrangements, how rearranged mtDNAs subsequently accumulate within cells, and the pathological consequences when this occurs.

## Introduction

Mitochondria are the major energy-transducing organelles of eukaryotic cells. The majority of cellular ATP is generated by the oxidative phosphorylation (OXPHOS) system, consisting of five multi-subunit enzyme complexes located at the inner mitochondrial membrane (IMM). Mitochondria originate from an ancient endosymbiotic merger between an alpha-proteobacterium and a host cell [1], and the genome of this bacterium (the proto-mitochondrion) persists in modern eukaryotic cells as mitochondrial DNA (mtDNA). Human mtDNA is a highly reduced circular, double-stranded, multicopy DNA genome that encodes many essential protein components of the OXPHOS system, as well as the RNA molecules required for the synthesis of these proteins within mitochondria [2–4].

The number of mitochondrial genomes per cell (referred to as copy number) is maintained through mtDNA replication, which occurs throughout the cell cycle in a process known as relaxed replication [5]. The replication of mtDNA relies on a nuclear-encoded protein machinery that is largely, but not completely, distinct from that in the nucleus. An impairment of mtDNA function, which can take the form of point mutations or deletions of segments of mtDNA, or as an inability to maintain enough mtDNA copies per cell, leads to a consequent impairment of OXPHOS function [6]. The multi-copy nature of mtDNA allows distinct mtDNA species, containing point mutations or rearrangements, to coexist in variable proportions in a state termed heteroplasmy [7]. The proportion of pathological mtDNA molecules determines whether mitochondria become dysfunctional enough to cause clinical disease [8,9] because wild-type mtDNA molecules can buffer the deleterious effects of pathogenic variants [10–13], and the proportion of mutation-containing mtDNA molecules must exceed a threshold value before causing OXPHOS deficiency at the cellular level [13].

Deletions within mtDNA can occur spontaneously but are also associated with mutations within genes that encode proteins required for mtDNA replication, as well as the related processes of mitochondrial nucleotide metabolism and mitochondrial dynamics [14,15]. This loss of mtDNA function manifests as a subset of mitochondrial diseases termed mtDNA maintenance disorders (MMD) [14,16,17].

## mtDNA structure and function

**Figure 1.**
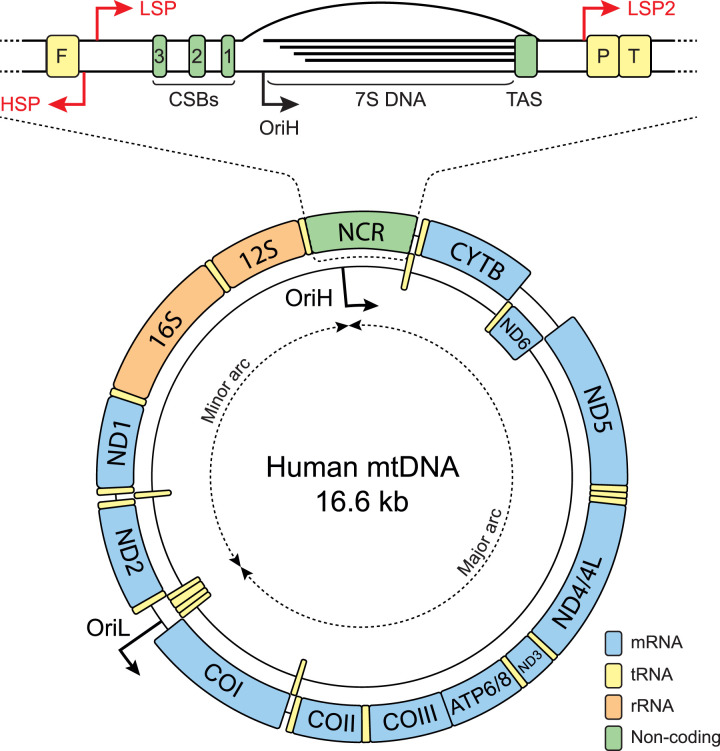
Human mtDNA structure. The positions of mtDNA-encoded genes are shown separated according to whether they are encoded by the heavy strand (H-strand) or light strand (L-strand), and the location of the replication origins OriH and OriL are indicated. An enlargement of the non-coding region (NCR, top) shows the location of the three promoters HSP, LSP and LSP2, as well conserved sequence blocks (CSBs) 1, 2 and 3, and 7S DNA, with its 5′ end in the OriH region and its 3′ end at the termination-associated sequence (TAS).

The human mitochondrial genome is a compact circular double-stranded DNA molecule of 16.6 kb. It encodes 13 proteins, all of which are components of OXPHOS complexes I, III, IV and V, as well as 22 tRNAs and 2 rRNAs (components of the small and large subunits of the mitochondrial ribosome) that are required for protein synthesis within the mitochondrial matrix (Figure 1). Polypeptides produced by translation at mitochondrial ribosomes are embedded directly into the IMM during assembly of the OXPHOS complexes [18–20].

mtDNA is highly compact, with the protein-coding genes containing no introns, and with two pairs of overlapping genes. Human mtDNA contains only one major non-coding region (NCR), which contains many of the sequence elements required for mtDNA replication and transcription (Figure 1) [21]. Genes are encoded by both strands of mtDNA, and transcription is polycistronic and covers most of the genome. Two promoters for mitochondrial transcription, the light-strand promoter (LSP) and heavy-strand promoter (HSP), are located close together in the NCR on opposite strands [22]. Most protein-coding genes are flanked by tRNAs, and endonucleolytic cleavage of the ends of these tRNAs by RNaseP [23] and ELAC2 [24,25] releases individual mRNAs, tRNAs and rRNAs for translation [26]. A second heavy-strand promoter (HSP2) was mapped using guanylyltransferase capping in early experiments [22]. However, discrepancies between the location of the HSP2 transcription start site *in vivo* and *in vitro*, and the apparent lack of a requirement for TFAM during initiation [22,27,28], has led to debate over the validity of HSP2 as a bona fide promoter [29]. The HSP2 site was also absent from recent high-throughput studies of human transcription initiation sites [30–32]. More recently a second promoter of the light strand was identified and named LSP2 [31]. LSP2 is located at the opposite end of the NCR from LSP, and upstream of all LSP-derived transcription products. This positioning suggests that LSP2 could be used for transcription of mtDNA-encoded genes, while LSP could be used primarily for mtDNA replication [29,31], but in-depth functional studies will be required to confirm or refute this idea.

mtDNA contains two canonical origins of mtDNA replication, termed OriH and OriL, with the intervening unequal regions being referred to as the major arc and the minor arc. OriH is located within the NCR, downstream of LSP [33], while OriL is located in a cluster of five tRNA genes approximately two-thirds of the distance around the genome, in the direction of mtDNA replication [34]. At any given time a proportion of mtDNA molecules contain a stable displacement loop (D-loop), formed by the incorporation of a third linear DNA strand of ∼650 nt called 7S DNA [35]. The D-loop region extends from the OriH region, at the 5′ end of 7S DNA, to the termination-associated sequence (TAS) at the 3′ end of 7S DNA, immediately upstream of the mt-tRNA^Pro^ gene [36]. The location and rapid turnover of the D-loop suggests that it represents a prematurely-terminated mtDNA replication intermediate, although alternative theories for the function of the D-loop have been proposed [37].

## The mtDNA replication machinery

### Primer formation

Mitochondrial transcription from LSP creates the primers for mtDNA synthesis from OriH, and as a result the core mitochondrial transcription machinery, consisting of the RNA polymerase POLRMT and the two transcription factors TFAM and TFB2M [38], can be considered as being essential for heavy-strand mtDNA replication initiation. Because TFB2M and TFAM are required for positioning the RNA polymerase and melting the promoter DNA during promoter-specific transcription initiation, they are not required for lagging-strand replication priming at OriL, for which the DNA template is already single stranded [38,39]. POLRMT is a single-subunit RNA polymerase related to T7 RNA polymerase (T7 RNAP) [40–42]. During promoter-specific transcription initiation, TFAM acts by binding upstream of the promoter, inducing a sharp bend in the DNA and recruiting POLRMT to the transcription start site [43,44]. TF2BM, and its paralogue TFB1M, were identified through their homology with the yeast mitochondrial transcription factor mtTFB, and show similarity with rRNA methyltransferases [38]. However, whereas TFB1M has retained this methyltransferase activity, and methylates two conserved adenine residues in the mitochondrial 12S rRNA [45,46], TFB2M has lost this activity and instead promotes melting of the promoter template during transcription initiation [47,48]. Primers synthesised from LSP require nucleolytic processing to generate RNA 3′ ends, hybridised to the DNA template, that can be used for replication initiation. RNASEH1 is a monomeric enzyme that specifically removes the RNA component of RNA:DNA hybrids [49], by processing [50] and removing the primers [51,52] synthesised by POLRMT. RNASEH1 possesses an N-terminal mitochondrial targeting sequence that directs import of the protein into mitochondria [53], and RNASEH1 activity is essential for mtDNA replication [54].

### Replication elongation

The minimal protein machinery capable of highly active and processive DNA synthesis using model substrates *in vitro* consists of POLγ (composed of POLγA and POLγB), TWINKLE, and MTSSB [55]. The loss of any one of these four proteins in mice results in embryonic lethality [32,56–58].

POLγ was one of the first human DNA polymerases to be identified [59], and remains the only known mitochondrial replicative DNA polymerase. The primase-polymerase PRIMPOL is documented to localise to mitochondria and is implicated in translesion synthesis and replication fork restart [60–62]. Other polymerases for which localisation to mitochondria has been suggested include DNA polymerases zeta [63,64], theta [65,66] and beta [67,68]. The possibility of these proteins localising to mitochondria has been reviewed in detail elsewhere [69], and as there is no documented role of these proteins in mtDNA replication elongation or mtDNA instability they are not discussed further. Mammalian POLγ is a heterotrimer comprised of a 140 kDa catalytic subunit, POLγA, and a dimeric 55 kDa accessory subunit, POLγB [70]. POLγA possesses both 5′→3′ polymerase and 3′→5′ exonuclease activities [71], while POLγB shows DNA binding activity and stimulates the processivity of the holoenzyme [72,73]. POLγA belongs to the family A of DNA polymerases, which also includes the *Escherichia coli* DNA polymerase I and bacteriophage T7 DNA polymerase (T7 DNAP) [74]. Although T7 DNAP and POLγ both utilise accessory factors that increase polymerase processivity, T7 DNAP co-opts the host thioredoxin for this role [75], while POLγB shows homology with aminoacyl tRNA synthetases [76,77], suggesting that POLγB may have been repurposed as an accessory factor.

The mitochondrial replicative helicase, TWINKLE, forms a hexameric complex that unwinds DNA in the 5′→3′ direction [78]. TWINKLE is homologous to the gp4 helicase-primase of bacteriophage T7 [79], although metazoan TWINKLE has lost key residues required for primase activity [80]. POLγ and TWINKLE together are capable of DNA synthesis *in vitro*, but this reaction is greatly stimulated by the mitochondrial single-stranded DNA (ssDNA) binding protein, MTSSB [55]. MTSSB is homologous to *E. coli* SSB [81], and forms a tetrameric complex that binds and wraps ssDNA [82–85]. MTSSB plays multifaceted roles in mtDNA maintenance and replication by being required for mtDNA replication initiation [32,50], protecting displaced template strands [86], directing sequence-specific priming of L-strand replication [32,86], and stimulating the activities of other replisome components [55,87,88].

### Replication termination

At the completion of mtDNA replication, nuclease activities are required to remove primers and DNA flaps, a ligase activity is required to seal the resulting nicks, and interlinked daughter molecules must be separated (decatenated). Current data supports the involvement of an RNA-specific nuclease (RNASEH1), a DNA-specific nuclease (MGME1) and the exonuclease proofreading activity of POLγ in replication termination [89]. MGME1 is a dedicated mitochondrial enzyme that belongs to the PD-(D/E)XK superfamily of nucleases, and is an ssDNA-specific exonuclease that requires a free DNA end to initiate catalysis [90,91]. The 3′→5′ exonuclease activity of POLγ also restrains the strand-displacement activity of POLγ during polymerisation and aids the formation of a ligatable nick [92]. Of the three ATP-dependent DNA ligases in human cells, LIG3 is targeted to both the nucleus and mitochondria [93], with the mitochondrial isoform being essential for cell viability [94,95].

The rotation of the replisome during DNA synthesis creates interlinked DNA molecules. These molecules can be decatenated by either type IA or type II topoisomerases, which operate using a strand-passage mechanism whereby a break is formed in ssDNA or dsDNA, an intact strand is passed through the break, and then the break is resealed [96]. The type IA topoisomerase TOP3A is a ssDNA decatenase related to bacterial Top3, and is targeted both to the nucleus and to mitochondria in human cells [97,98]. *TOP3A* is an essential gene [99], and the loss of the mitochondrial isoform of TOP3A leads to the accumulation of hemicatenated mtDNA replication products [100]. The type II topoisomerases TOP2A and TOP2B also utilise a strand-passage mechanism and could theoretically also participate in mtDNA decatenation, although their genetic knockout does not affect the topological state of mtDNA [88,100] and there are conflicting reports about their localisation [88,100–104].

## Mechanisms of mtDNA replication

This section will cover basic details of replication initiation from the two canonical origins of mtDNA replication, OriH and OriL, as well as aspects of replication termination and DNA topology, before discussing the differing proposed modes of the mechanism of mtDNA replication.

### Replication initiation at OriH

**Figure 2.**
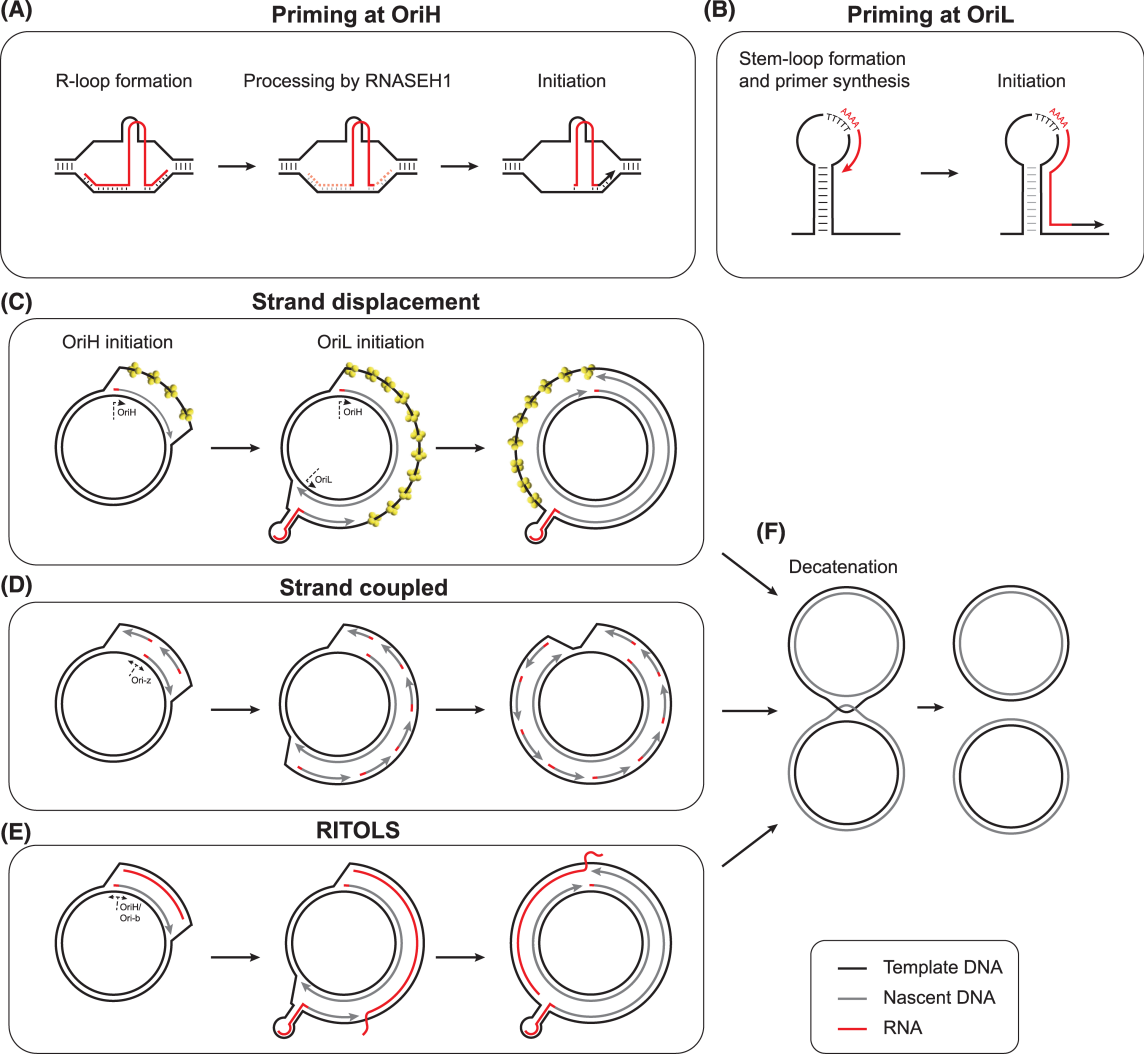
Mechanisms of mtDNA replication. (**A**) Replication priming in the OriH region. Transcription from LSP by POLRMT creates a hybrid R-loop anchored at CSB2, which is processed by RNASEH1 to generate a 3′ RNA end that can be utilised by POLγ for DNA synthesis. (**B**) Replication priming at OriL. When exposed in ssDNA form, the OriL sequence forms a stem-loop structure. POLRMT initiates RNA synthesis from a poly(T) stretch in the loop of this structure, generating a primer for DNA synthesis by POLγ. (**C–E**) Models of mtDNA replication. (**C**) The strand displacement model (SDM). Leading (heavy) strand replication is initiated at OriH, and the displaced lagging strand template is coated with MTSSB (yellow). Lagging strand replication is initiated by POLRMT, which forms a primer at a stem-loop structure at the OriL site, allowing continuous L-strand synthesis. (**D**) Strand coupled replication. Initiation is associated with a broad zone downstream of the NCR, Ori-z. Replication of the H-strand is continuous, while L-strand replication requires the formation of discontinuous Okazaki fragments. (**E**) Ribonucleotide incorporation throughout the lagging strand (RITOLS). Replication is initiated in the NCR, with OriL being the major site of lagging-strand DNA synthesis. The displaced lagging-strand template is coated with RNA transcripts (bootlaces) rather than MTSSB. (**F**) Hemicatenated mtDNA replication products are separated by TOP3A.

The location of LSP upstream of the OriH site, as well as the discovery of RNA covalently attached to the 5′ ends of DNA molecules in the OriH region [33,105], first indicated that transcription initiation from LSP could generate the primers for mtDNA replication initiation from OriH, as well as for the synthesis of 7S DNA (Figure 2A). The initiation of RNA synthesis from LSP minimally requires POLRMT, TFAM, and TFB2M; the mechanism and regulation of mitochondrial transcription has recently been reviewed in detail elsewhere [29]. Premature termination of transcription within the NCR can either produce primers for mtDNA replication or a free polyadenylated RNA species called 7S RNA [106], which has recently been found to interact with POLRMT to regulate transcription activity [107]. To be utilised as primers, RNA products from LSP must form R-loops, in which the primer RNA remains stably bound to the template DNA, displacing the non-template strand. The Conserved Sequence Box (CSB) sequences between LSP and OriH, particularly CSB3 and CSB2, are required to anchor the formation of R-loops [50,108,109], which also involves hybridisation with the displaced H-strand [110]. Attempts to reconstitute mtDNA replication initiation from OriH have found that these R-loops require additional nucleolytic processing before they can be utilised by POLγ. This processing activity was originally proposed to be provided by the ribozyme RNase MRP [111–113], but questions over the mitochondrial localisation of the catalytic RNA molecule of this complex [114,115] and the lack of a mechanism to import the RNA into mitochondria [116], later cast doubt on this hypothesis. More recently, RNASEH1 has been found to be able to provide the R-loop processing activity required for primer formation [50].

Once the primer has been processed, POLγ is able to synthesise the nascent H-strand, which either terminates at TAS (to form 7S DNA) or proceeds through the TAS region to replicate the full length mtDNA. The rate of replication initiation from OriH could therefore conceivably be controlled by the rate of primer formation from LSP, or by whether or not DNA synthesis terminates at TAS. The majority of DNA synthesis events from OriH terminate with the formation of 7S DNA, even in cells actively amplifying mtDNA copy number [117,118], but how this termination occurs remains poorly understood [37].

*In vitro*, transcription from OriH by POLRMT frequently terminates at the CSB2 site [119–121]. The addition of the transcription elongation factor TEFM, which increases the affinity of POLRMT for the DNA template and promotes the formation of long transcription products [120,122], also promotes transcription through CSB2 [119,120]. The presence or absence of TEFM has therefore been suggested to act as a determining factor between primer formation and full-length transcription [119]. A recent mouse knockout of TEFM exhibited increased mtDNA copy number, but transcription initiation from LSP did not typically reach the CSB sites [123], arguing against a simple molecular switch mechanism. Alternatively, TEFM could bind to all elongating transcription complexes, and therefore represent an essential component of the mitochondrial transcription machinery.

### L-strand replication priming at OriL

Early studies of mtDNA replication observed firstly that leading-strand replication from OriH must pass the OriL site before lagging-strand replication from OriL can be initiated [124], and secondly that the OriL template sequence has the ability to form a strong stem-loop structure with a stretch of poly(T) residues in the loop [34,36]. These observations were later explained by the discovery that POLRMT can bind to this stem-loop structure and initiate non-processive transcription from the poly(T) stretch to generate the primer for L-strand synthesis from OriL (Figure 2B) [125–127]. Replication of the H-strand must therefore pass the OriL site to displace the lagging-strand template in single-stranded form, which allows the OriL sequence to adopt this secondary structure. The stem-loop structure of OriL firstly ensures that the poly(T) stretch used for primer synthesis is available in single-stranded form [125,128], and secondly prevents MTSSB from binding to, and blocking, the initiation site [32,86]. The OriL sequence is both conserved and refractory to mutation or deletion *in vivo* [128,129], indicating that OriL is essential for mtDNA replication, as mtDNA molecules without a functional OriL sequence cannot be propagated.

### mtDNA topology during mtDNA replication

Moving polymerase complexes create regions of positive DNA supercoiling (overwinding) ahead of the complex and regions of negative DNA supercoiling (underwinding) behind the complex [130]. Unresolved supercoiling can inhibit replication fork progression and promote the formation of R-loops [96]. Additionally, the supercoiling of replicating DNA can drive the formation of crossovers between the replicating molecules that must be removed before the replicated molecules can be separated [131]. These problems are solved by topoisomerases, which alter DNA topology by creating transient breaks in the DNA backbone [96]. Mitochondria possess a dedicated type IB topoisomerase, TOP1MT, which can remove both positive and negative supercoiling [132]. Although TOP1MT has primarily been implicated in mitochondrial transcription [133,134], binding sites for TOP1MT have also been mapped to sites important for mtDNA replication [135,136], its loss leads to mtDNA replication stalling [88], and its activity is stimulated by MTSSB [88], suggesting that TOP1MT also contributes to the maintenance of mtDNA topology during replication. In addition to TOP1MT, the type IA topoisomerase TOP3A localises both to mitochondria and the nucleus in human cells [97,100]. TOP3A operates using a strand passage mechanism that allows it to both remove negative supercoiling and decatenate interlinked molecules containing regions of ssDNA [98,137,138]. This decatenation activity of TOP3A is required to separate replicated mtDNA molecules (Figure 2F) [88,100,139], and loss of mitochondrial TOP3A leads to the formation of replicated mtDNA molecules containing hemicatenanes close to OriH [100]. *In vivo*, TOP3A may also be able to remove interlinks between replicating molecules while replication is still proceeding, so that the completion of H-strand synthesis is concomitant with segregation [104,139].

### Replication termination and primer processing

For mtDNA replication to be completed, the RNA primers must be removed, and the DNA strands ligated to form covalently closed molecules. RNASEH1, which specifically removes the RNA component of RNA:DNA hybrids, is able to remove the majority of the RNA primer in mitochondria, leaving ∼1–3 nt of RNA [51,140]. RNASEH1 is essential for mtDNA replication [52,54], and loss of RNASEH1 activity results in retention of primer RNA at replication origins that inhibits further DNA replication and leads to mtDNA breakage [51,52].

POLγ, like other DNA polymerases, possesses a limited ability to displace an upstream 5′ DNA end into a short flap structure, and to co-operate with nucleases to remove this flap in order to generate a ligatable nick [92]. If no RNA remains attached to the 5′ end of the DNA strand, short DNA flaps produced by POLγ can be removed by MGME1 to leave a nick that can be sealed by LIG3 [141]. However, MGME1 is inefficient at processing RNA-containing flaps [90] and in this case an additional flap endonuclease activity, such as that possessed by FEN1, is required to create a ligatable nick [140].

### Strand displacement replication

The strand displacement model (SDM) was first proposed based on transmission electron microscopy (TEM) data of replicating mtDNA [124] isolated using CsCl density gradient centrifugation, which was originally developed for the analysis of mtDNA [142]. The SDM remains the most widely accepted mechanism of mtDNA replication (Figure 2C). It posits that OriH and OriL are the major (or sole) origins for mtDNA synthesis, and that replication from OriH must precede replication from OriL. During the substantial delay between the initiation of H-strand synthesis from OriH and the initiation of L-strand synthesis from OriL, the displaced L-strand is coated and protected by MTSSB [86]. MTSSB is abundant enough within mitochondria to fully coat the L-strand [86,143] and the binding pattern of MTSSB predicted by the SDM has also been corroborated *in vivo* using ChIP-seq [86]. The discontinuous nature of SDM replication means that DNA template unwinding by TWINKLE is required for H-strand replication from OriH, but not for L-strand replication from OriL, for which the template is already single-stranded. Because synthesis of the two mtDNA strands is unidirectional and initiated from distinct sites, synthesis of the nascent H-strand is expected to be completed before synthesis of the L-strand [124,144,145]. Replicated mtDNA segregates as open circular molecules and is subsequently converted into closed circular supercoiled molecules [145]. The core principles of the SDM have been supported by many studies that have biochemically reconstituted replication initiation from both OriH and OriL [50,126,127], and shown that OriL is both essential and the principal origin of lagging-strand replication [125,128].

### Strand-coupled replication

The application of 2-dimensional agarose gel electrophoresis (2D-AGE) to replicating mtDNA molecules allowed the visualisation of fully double-stranded mtDNA replication intermediates, characteristic of coupled leading- and lagging-strand DNA replication, throughout the mtDNA [146]. Such intermediates would not be expected in the case that OriH and OriL are the sole replication origins, for which the L-strand template would be predominantly single stranded (Figure 2D). Double-stranded mtDNA replication intermediates have also been visualised in TEM and atomic force microscopy preparations [147,148]. Bidirectional replication initiation events, associated with duplex replication intermediates, were mapped to a broad zone downstream of the NCR, termed Ori-z [149]. Replication initiation events in this region have also been seen in cases where mtDNA topology is severely dysregulated [88]. It is worth noting that there is no indication that the polymerase complexes for H- and L-strand mtDNA replication physically or functionally interact, so it is possible that the existence of additional lagging-strand origins of replication, that are used less frequently than OriL, could account for the presence of double-stranded replication intermediates [150].

### RITOLS replication

The RITOLS model, short for Ribonucleotide Incorporation Throughout the Lagging Strand, was developed based on mtDNA replication intermediates visible by 2D-AGE that are resistant to cleavage using standard restriction enzymes but that are sensitive to RNASEH1. These intermediates were found to contain RNA annealed specifically to the lagging-strand template during replication [151,152]. This RNA was later suggested to derive from processed mitochondrial transcripts that can anneal to the lagging strand template and were termed bootlaces (Figure 2E) [153,154]. The RITOLS model otherwise shares many features with the SDM, including the asynchronous nature of replication and the use of the OriH region and OriL as the principal sites of DNA synthesis. An additional bidirectional replication origin, Ori-b, was mapped to the NCR downstream of OriH and associated with RITOLS intermediates [152,155]. The apparently unidirectional nature of mtDNA replication [152,156] would imply that, if bidirectional replication initiation were to occur in the NCR, then one of the replication forks must become arrested at OriH, while the other proceeds around the major arc [155]. The RITOLS model has struggled to gain widespread acceptance, due in part to a lack of mechanistic clarity over how the processed and structured mitochondrial transcripts would be annealed to the lagging-strand template, the presence and essential nature of mitochondrial RNASEH1 activity [51,52,54], and the difficult and esoteric nature of 2D-AGE data interpretation [157].

## Mechanisms of mtDNA instability

### Deletion classes and spectra

Deletions of mtDNA are classified according to the presence and type of repeat sequences at the deletion breakpoints. Class I deletions, which occur at direct repeats, typically constitute 65–75% of all reported breakpoints, with the remaining deletions either showing imperfect homology at the breakpoint (Class II) or no detectable homology (Class III) [158–166].

**Figure 3.**
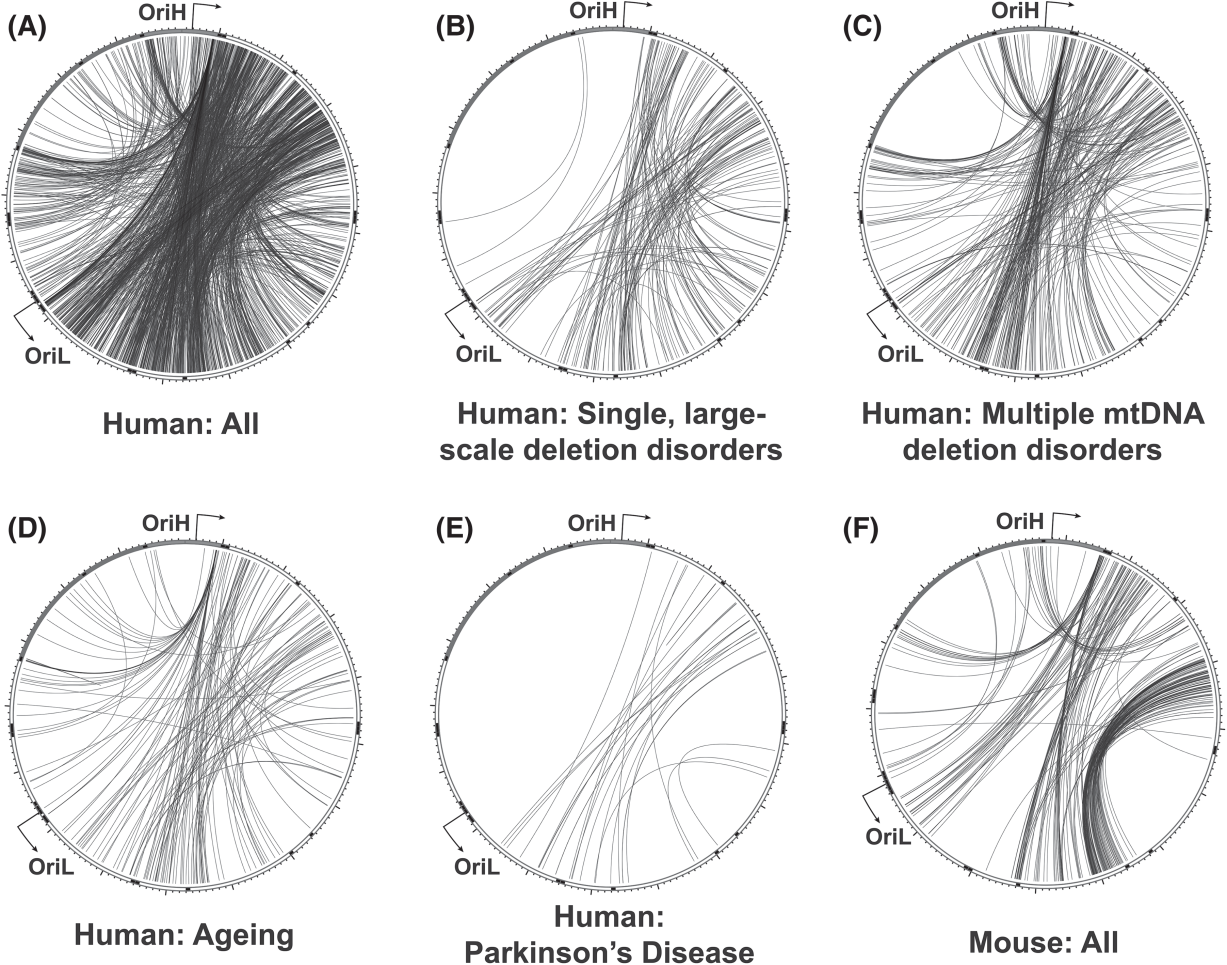
Rearrangement breakpoint patterns in mammalian mtDNA. All reported human mtDNA breakpoints in the MitoBreak database [168] were plotted using Circos (**A**), or categorised by association with single, large-scale deletion disorders (**B**), multiple mtDNA deletion disorders (**C**), ageing (**D**), or Parkinson's disease (**E**). All reported breakpoints in mouse mtDNA (**F**) are shown for comparison. The outer track indicates the locations of mitochondrial mRNAs (white), tRNAs (black), rRNAs (dark grey) and the NCR (light grey).

The vast majority of mtDNA deletions are contained within the major arc, between the replication origins OriH and OriL [167,168]. The origins of mtDNA replication must be preserved in deletion-containing molecules for the deletion to be propagated [128,129]. The location of replication origins, and likely the mechanism of mtDNA replication, therefore place constraints on when and where deletions can form (Figure 3). This means that although the majority of mtDNA deletions are associated with direct repeats, most direct repeats are not associated with deletions. It should also be noted that many methods for mapping mtDNA breakpoints do not distinguish between deletions and duplications, and so some breakpoints, most obviously those that appear to remove an origin of replication, are likely to constitute duplications. The most widely studied Class I deletion, termed the common deletion, removes 4977 bp of the major arc between two 13 bp direct repeats. This rearrangement is frequently seen in sporadic cases of mitochondrial disease associated with single, large-scale deletions (SLSD) [169,170], as well as in multiple deletion disorders [163,171] and ageing [172–177]. Of the five direct repeats of 13 bp or greater in human mtDNA, the common deletion is both the only one frequently observed *in vivo*, and the only one for which both repeats are located in the major arc [158,169].

Deletions associated with repeat sequences typically remove one of the two repeats. Imperfect repeats can therefore be used to assign directionality to the rearrangement, according to which repeat is retained and which is lost. Analyses of these breakpoints have found that most Class II deletions either retain the 5′ repeat sequence or occur within the repeat, with removal of the 5′ repeat being rarer [158,163,164].

An additional ‘hotspot’ for deletion formation is found in the TAS region, and deletions between this site and multiple sites in the major arc are frequently seen in patients with multiple mtDNA deletions [129,163,178,179]. These deletions typically show little or no homology at the breakpoints, and their formation may instead relate to the triple-stranded structure of mtDNA at this site showing a greater propensity for replication slippage or breakage [37].

### Deletion formation during mtDNA replication

Models for the formation of mtDNA rearrangements have suggested that deletions are either formed during mtDNA replication or driven by the presence of double-strand breaks, although these mechanisms are not mutually exclusive.

**Figure 4.**
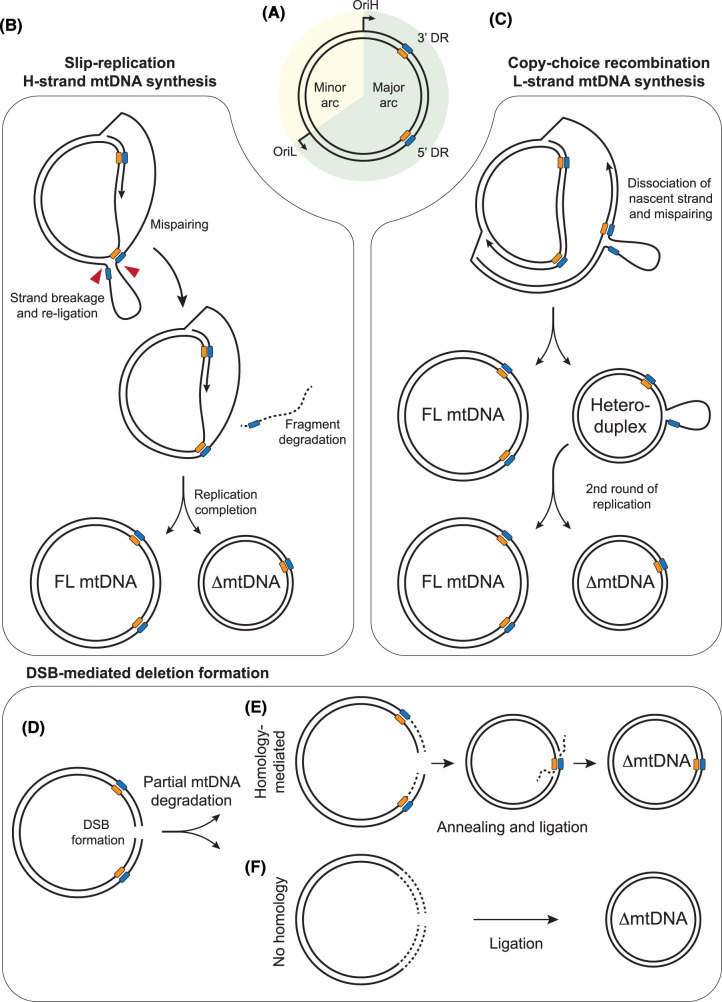
Mechanisms of mtDNA deletion formation. (**A**) Representation of mtDNA, containing direct repeat (DR) sequences (blue and orange boxes) in the major arc. (**B**) Slip-replication model. Mispairing of an upstream repeat sequence in the displaced lagging-strand template, followed by breakage of the displaced loop (red arrows), generates a truncated template molecule that is replicated to generate a deletion-containing mtDNA. (**C**) Copy-choice recombination model. Slippage and mispairing during lagging-strand mtDNA replication forms a heteroduplex molecule containing a single-stranded loop. Replication of this molecule generates one full-length mtDNA molecule and one deletion-containing molecule. (**D–F**) Models of mtDNA deletion formation associated with strand breaks. Generation of a double-strand break (DSB) in mtDNA (**D**), followed by partial degradation of the broken DNA ends, creates truncated mtDNA molecules. Pathways are shown both for homology-mediated annealing of resected DNA ends (**E**) and the ligation of partially-degraded DNA ends without homology (**F**).

Several lines of evidence support the idea that mtDNA deletions, particularly those associated with repeats (Figure 4A), can be formed during mtDNA replication. First, the directionality of deletions at imperfect repeats [158,163,164] implies a slippage mechanism during DNA synthesis, as deletions associated with DNA breakage would be expected to be symmetrical regarding repeat sequences. Second, the pattern of deletions formed during DNA synthesis by POLγ *in vitro* closely resembles that seen in clinical samples [163]. Third, the clustering of deletions in the major arc, between the origins of DNA replication, suggests a link to the mechanism of mtDNA replication, although the minor arc contains relatively fewer direct repeats [180]. Fourth, deletion breakpoints cluster around sequences that are expected to be difficult to replicate, such as homopolymeric tracts [171,181] and predicted G4 quadruplex structures [182,183], which have recently been observed to form *in vivo* [184].

An early model for the mechanism of mtDNA deletion formation, termed slip-replication, was originally suggested to explain the formation of the common deletion [170]. This model suggests that during H-strand synthesis (leading-strand replication) a repeat from the displaced parental H-strand anneals with a downstream repeat on the leading strand template. A break would then need to be created in the displaced loop of parental H-strand ssDNA, and then re-ligated, after which a deletion-containing molecule would be formed during lagging-strand synthesis [170] (Figure 4B).

More recently an alternative model, copy-choice recombination, has proposed instead that deletions are formed during synthesis of the mtDNA L-strand, corresponding to lagging-strand synthesis from OriL (Figure 4C). According to this model, the dissociation of the replisome and slippage of the nascent lagging strand to a downstream repeat sequence on the template causes the skipping of the intervening sequence. The newly replicated truncated molecule will be converted into a fully dsDNA deletion-containing molecule following a second round of replication [163]. Slippage during lagging-strand replication requires the lagging-strand template to be in a single-stranded conformation, as predicted by the strand-displacement model of mtDNA replication. The copy-choice recombination model is supported by biochemical reconstitution experiments and matches the observed tendency towards retention of the 5′ repeat sequence during the formation of Class II deletions [163]. Furthermore, the creation of nicks immediately downstream of the 5′ repeat *in vivo*, which would be expected to promote the slippage of the template, has been found to increase the rate of deletion formation between repeats [185], consistent with this model. A similar mechanism of deletion formation has been documented in *E. coli* [186]. Such a mechanism would imply that any factors that cause stalling of the mitochondrial replisome would promote template slippage and consequently deletion formation [181]. This could therefore explain why defects in factors involved in distinct pathways can all result in similar patterns of deletion breakpoints (Figure 3) [14]. In this model, replication stalling induced either by mutations in components of the mtDNA replication machinery, by a deficiency or imbalance in mitochondrial nucleotide levels, or by a lack of content mixing resulting from impaired mitochondrial fusion, all result in mtDNA replication stalling that promotes template slippage. Deletion formation at specific repeats could be made more likely by the sequences being brought into proximity by secondary structures [163] or by extended regions of imperfect homology [162].

### mtDNA deletions associated with DNA breakage

Several studies have found that the creation of a large number of double-strand breaks in mtDNA promotes the formation of deletions (Figure 4D). By engineering either cells or mouse models in which a restriction endonuclease is targeted for import into mitochondria, site-specific double-strand breaks have been induced in mtDNA, leading to a rapid loss of mtDNA copy number [187–193]. Following recovery of copy number, rearrangement-containing molecules can be detected, with breakpoints frequently clustered around the restriction site and the TAS region [188,189,191–193].

Deletion formation associated with mtDNA strand breaks has been suggested to occur via aberrant repair of linear mtDNA molecules [180,194,195]. This could occur through the annealing of short regions of homology following limited nucleolytic processing (resection) of double-stranded ends, or by simple ligation of broken molecules. In the nucleus, double-strand breaks would typically be repaired either by non-homologous end joining or by homologous recombination (HR), both of which require DNA binding factors and enzymatic activities that have not been found in mitochondria [194,196,197]. Furthermore, the use of HR leads to the exchange of sequence information between molecules, where homologous sequences have been used as a template for DNA synthesis across the break site [198]. In a study of mice harbouring two stable heteroplasmic variants, no exchange of sequence information was found to have occurred after more than fifty generations, arguing against the existence of a homology-directed repair mechanism in mitochondria [196]. Nevertheless, in the presence of high levels of linear mtDNA molecules, deletions could conceivably be formed by the undirected annealing of partial degradation products, or by the ligation of broken mtDNA ends by LIG3 [195]. In this case the rapid degradation of linearised mtDNA molecules would be important to prevent deletion formation [189].

Interestingly, many of the deletions observed following mtDNA cleavage with targeted restriction enzymes show little homology at breakpoint sites [188,189,191,192] compared with deletions seen in human pathologies, most of which occur at direct repeat sequences [167,168]. However, breakpoints at the TAS site, which is often seen as a hotspot for deletion formation in patients with multiple mtDNA deletions in MMD [171,178,179], also frequently show little homology, providing support to the idea that mtDNA strand breaks may be relevant for deletion formation under normal *in vivo* conditions. Double-strand breaks in mtDNA could conceivably form via DNA replication stress, or as the result of exogenous agents such as chemotherapeutics or radiation. Further insights into the *in vivo* rate of mtDNA double-strand break formation, and the mechanism of mtDNA degradation, would help to establish whether mtDNA strand breaks are a common cause of mtDNA deletions (particularly Class III deletions) in addition to situations in which high levels of strand break have been artificially induced.

### Stable linear mtDNA molecules

As noted above, linear mtDNA molecules are rapidly degraded under normal conditions and so are not usually detectable [187–193]. However, in certain conditions a long linear mtDNA fragment corresponding to the entire major arc, with ends at the replication origins OriH and OriL, can be seen. As this molecule lacks complete origins it would not be expected to be replication competent, and its ligation would form a circular deletion-containing molecule that also could not be propagated, indicating that it must constantly be produced by *de novo* mtDNA replication. This linear species was first observed in the mutator mouse, which lacks the 3′-5′ exonuclease activity of POLγ [199,200]. Similar linear molecules were subsequently seen in patients [201] and in mice [202] lacking the mitochondrial matrix exonuclease MGME1, as well as *Rnaseh1* knockout mice [52]. Mouse models of all three of these cases display site-specific mtDNA replication stalling at both origins of replication [52,200,202], suggesting that the linear species could be produced by breakage of mtDNA replication intermediates at fragile sites [200]. However, all of these factors also participate in the removal of primers and the generation of ligatable ends at OriH and OriL [51,92,140,141]. An alternative model suggests that these linear mtDNA molecules are generated via replication of mtDNA molecules that contain persistent nicks at the replication origins, because of defective primer- and flap-processing activities [92]. Consistent with this model, the loss of the mitochondrial topoisomerases TOP3A and TOP1MT also causes the accumulation of mtDNA replication intermediates stalled around the replication origins, but without causing spontaneous breakage and the generation of linear mtDNA fragments [88].

### mtDNA duplications

Partial duplications of mtDNA have been reported in some circumstances, although these are rare in comparison with deletions. High levels of specific single duplications have been seen in sporadic cases of mitochondrial disease, particularly associated with diabetes [203–205]. Duplications within mtDNA, associated with nuclear DNA variants in genes involved in mtDNA maintenance, have also been observed. A notable example is the mtDNA mutator mouse, which shows a complex and tissue-specific pattern of duplications within the NCR termed control region multimers [206]. Patients with loss-of-function mutations in *MGME1* have also been found to harbour multiple mtDNA duplications in muscle, again concentrated around the NCR region, although frequently duplicating the entire NCR and adjacent genes [201]. The similarities between these two cases may also point to a link between defective mtDNA flap processing and duplication formation.

An additional consideration when analysing mtDNA rearrangements is that duplications cannot be distinguished from deletions using only the breakpoints seen in short-read sequencing datasets [129]. It is therefore possible that the rate of duplication formation is higher than currently appreciated, particularly where deletions have been reported that apparently remove an essential origin of replication.

## Heteroplasmy and mtDNA inheritance

There has been a lot of recent interest in the transmission and selection of mtDNA variants in the literature (see reviews in [8,207–209]). Though much of the work has been focused on substitutions rather than major deletions in the organellar genomes, the models are broadly applicable to both. However, only the smallest, and most precise codon excising deletions could possibly be viewed as functionally inert to the cell. Most deletions span multiple kilobases, and truncate protein-coding genes, delete tRNA genes [210–212], and potentially create novel open reading frames [213]. It appears that such a fused open reading frame between two mitochondrial genes is the basis of mitochondrial male sterility in plants [214]. So how does a mutation that is most certainly deleterious to the cell survive and propagate within an organism?

Due to the multicopy nature of mtDNA in most cell types, any new rearrangement or mutation will be rare within the cell's mtDNA pool. As such, these mutations will initially be of little functional consequence to the cell. However, as mitochondria do not have a co-ordinated mechanism to ensure equal replication and segregation of each nucleoid, it is possible that this new mutation can either be lost during mtDNA turnover, or become more frequent in the mtDNA pool of that cell through various cellular mechanisms [8,208]. In addition, there has been a longstanding argument that shorter mtDNA molecules bearing deletions would take less time to replicate, so could potentially have a replicative advantage to aid in their propagation in the cell [215,216]. If the mutation occurs in a terminally differentiated cell later in life, such as an accumulated somatic mutation during the ageing process, it will remain isolated in that cell. However, mutations occurring during embryogenesis, growth, or in stem cell compartments may be propagated by cell proliferation, enhancing their pathogenic effects as the proportion of affected cells increases within tissues and across organ systems. Moreover, if occurring in germline cells, mutations could also be inherited by the organism through sexual reproduction. A more detailed discussion of the mechanisms of segregation can be found in a later section.

If a mutation becomes more prevalent in the cell, it becomes more likely that the biochemical consequence of the mutation is able to affect the cell, leading to altered function of the mitochondrial respiratory chain, or a change in exerted selection pressure for or against the mitochondria bearing a specific mutation. This phenomenon was first described in yeast and cell culture experiments, where cells were selected for mutations that conferred resistance to antibiotics such as chloramphenicol, antimycin or oligomycin [217], but was soon applied to patients with mtDNA-related disease and their families. This found that family members with low to moderate mtDNA mutation levels remained resistant to mitochondrial disease phenotypes, while those with a mutation-dependent ‘high’ level were afflicted with the mitochondrial disorder [13].

The first major recognisable pattern in the observed accumulated mtDNA deletions is the conservation of the control region and OriL regions in deletion-containing mtDNA molecules [164,165,171]. Even with the added depth of long-read detection studies, this pattern is so far maintained; deletions that remove OriL or the replication-associated regions of the control region remain rare observations, even in ageing tissues [218–220]. One assumes that this is due to the importance of these sites for the ability of the deletion-containing mtDNA to undergo strand-uncoupled replication. According to this hypothesis, the rare deletions would be incapable of replication, and so would remain as rare ‘one-off’ molecules. Thus, selection against mutations that incapacitate mtDNA replication in somatic tissues have long been acknowledged. The level of the deletions in tissues have also implied that somatic selection on these molecules has occurred. Studies of deletions associated with Pearson's syndrome have shown that, in some tissues, there appears to be a selective loss of the deletions, such as in the bone marrow or peripheral blood [221–223].

Thus, it is unsurprising that mtDNA deletions experience purifying selection in the germline, given that reading frame-disrupting mutations [207,224] or even rare amino acid substitution mutations [225] are known to experience purifying selection in animal germlines [226]. In the case of Pearson's syndrome, transmission of the deletion from affected mothers has been estimated at around 1 in 24 births, with the risk of recurrence being 1–9 in 117 births [227]. However, data from animal models imply that in specific circumstances, reliable transmission of deletions can occur [228]. A mouse line carrying a single, large-scale mtDNA deletion (ΔmtDNA^4696^) was reported in 2000, derived from mitochondria isolated from synaptosomal fragments from aged mouse brains which were transferred into ρ^0^ cells (cells lacking mtDNA), then fused into mouse embryos to generate the line [228]. For the first three generations, the authors reported the presence of a 27.6 kb, partially-duplicated mtDNA molecule. However, the partial duplication was rare, and was eventually lost, but the strain continued to faithfully transmit the ΔmtDNA^4696^ molecule.

## Clinical consequences of mtDNA instability

Mitochondrial diseases are a diverse group of inherited metabolic disorders that ultimately impair mitochondrial bioenergetics [6,229,230]. A subset of mitochondrial diseases are linked to mtDNA instability, causing either depletion of mtDNA copy number or mtDNA mutations, including deletions, such as cases of mtDNA SLSD [159,169,210,231] and MMD [14,16,230,232]. A summary of genes and clinical phenotypes associated with mtDNA deletions and depletion is provided in Table 1. SLSD arise early in development and accumulate over time causing disease [159,231,305,306], however, as described above transmission is rare and SLSD are considered sporadic [227]. In MMD, autosomal dominant or recessive variants disrupt mtDNA maintenance pathways (including mtDNA replication) and lead either to mtDNA depletion [276,307] or the accumulation of multiple mtDNA deletions within cells, causing disease [14,17,308]. In the North East of England, mitochondrial diseases affect ∼1 in 8000 adults (>16 years old), with 12% being SLSD and 22% due to a wide range of nuclear variants, 59% of which are MMD cases [242], while SLSD represented ∼20% of all cases (32% of cases of pathogenic mtDNA mutations) in an Italian cohort [233].

**Table 1.** Summary of human diseases, syndromes and phenotypes associated with genetic defects characterised by mitochondrial DNA (mtDNA) deletions or depletion

Gene	Disease/syndrome/phenotype	References
**Single, large-scale deletions (mitochondrial DNA defect)**		
Primary mitochondrial DNA (mtDNA) deletions	Pearson syndrome (PS)	[231,233–241]
	Kearns-Sayre syndrome (KSS)	
	Chronic progressive external ophthalmoplegia (CPEO)	
**Mitochondrial DNA maintenance disorders (autosomal defects)**		
***Mitochondrial DNA replication***		
*POLG*	Alpers-Huttenlocher syndrome *(mtDNA depletion syndrome)*	[242–252]
	Childhood myocerebrohepatopathy spectrum (MCHS) *(mtDNA depletion syndrome)*	
	Mitochondrial neurogastrointestinal encephalopathy (MNGIE)-type *(mtDNA depletion syndrome)*	
	Myoclonic epilepsy, myopathy, and sensory ataxia (MEMSA)	
	• Spinocerebellar ataxia with epilepsy (SCAE)	
	Ataxia neuropathy spectrum (ANS)	
	• Mitochondrial recessive ataxia syndrome (MIRAS)	
	• Sensory ataxia, neuropathy, dysarthria and ophthalmoplegia (SANDO)	
	Progressive external ophthalmoplegia (PEO)	
	• Autosomal recessive PEO (arPEO)	
	• Autosomal dominant PEO (adPEO)	
	Other phenotypes	
	• Charcot-Marie-Tooth (CMT) 2 type phenotype	
	• Parkinsonian phenotype	
*POLG2*	Autosomal dominant PEO (adPEO)	[253–256]
	Mitochondrial DNA depletion syndromes *(hepatic and neuro-ophthalmic types)*	
*TWNK*	Autosomal dominant PEO (adPEO)	
	Mitochondrial DNA depletion syndrome *(hepatocerebral)* or infantile-onset spinocerebellar ataxia (IOSCA)	
	Perrault syndrome	
*RNASEH1*	Autosomal recessive PEO (arPEO)	[250,257,258]
	Encephalomyopathic phenotypes	
*TOP3A*	Autosomal recessive PEO (arPEO)	[179,259–262]
	Bloom syndrome-like disorder *(mtDNA depletion syndrome)*	
*MGME1*	Mitochondrial DNA depletion syndrome *(multisystemic)*	[90,263]
	PEO phenotype (often cerebellar ataxia and profound emaciation)	
*DNA2*	Autosomal dominant PEO (adPEO)	[264–266]
	Seckel syndrome (genome instability)	
*TFAM*	Mitochondrial DNA depletion syndrome *(hepatocerebral)*	[267]
***Deoxyribonucleotide triphosphate (dNTP) metabolism***		
*RRM2B*	Autosomal dominant PEO (adPEO)	[232,268,269]
	Mitochondrial neurogastrointestinal encephalopathy (MNGIE)-type *(mtDNA depletion syndrome)*	
	Mitochondrial DNA depletion syndrome *(encephalomyopathic type with renal tubulopathy)*	
	Rod-cone dystrophy, sensorineural deafness, and fanconi-type renal dysfunction	
*TYMP*	Mitochondrial neurogastrointestinal encephalopathy (MNGIE) *(mtDNA depletion syndrome)*	[270–272]
	PEO phenotype (often milder gastrointestinal disease)	
*TK2*	Mitochondrial DNA depletion syndrome *(myopathic)*	[273–275]
	Autosomal recessive PEO (arPEO)	
*DGUOK*	Autosomal recessive PEO (arPEO)	[248,249,276–280]
	Mitochondrial DNA depletion syndrome *(hepatocerebral)*	
*MPV17*	Charcot-Marie-Tooth (CMT) disease, axonal, type 2EE	[248,249,276,277]
	Mitochondrial DNA depletion syndrome *(hepatocerebral)*	
*SUCLA2*	Mitochondrial DNA depletion syndrome *(encephalomyopathic)*	[281,282]
*SUCLG1*	Mitochondrial DNA depletion syndrome *(encephalomyopathic)*	[282,283]
*ABAT*	GABA-transaminase deficiency *(encephalopathic, mtDNA depletion syndrome)*	[284–286]
*SLC25A4*	Autosomal dominant PEO (adPEO)	[287–289]
	Mitochondrial DNA depletion syndrome *(cardiomyopathic)*	
*AGK*	Sengers syndrome	[290–292]
	Congenital cataract	
***Mitochondrial dynamics***		
*OPA1*	Autosomal dominant optic atrophy 1	[293–295]
	Autosomal dominant optic atrophy plus (variable myopathy, ataxia, and spasticity)	
	Mitochondrial DNA depletion syndrome *(encephalocardiomyopathic)*	
*MFN2*	Charcot-Marie-Tooth (CMT) disease (several types)	[296–301]
	Multiple symmetric lipomatosis (with or without neuropathy)	
*FBXL4*	Mitochondrial DNA depletion syndrome *(encephalocardiomyopathic)*	[302–304]

This is not intended to be an exhaustive list of all previously described features associated with each gene, and the full clinical spectrum for many of these mitochondrial diseases is likely to expand and require re-organisation as more variants with distinct phenotypes are identified.

### Clinical spectrum of mitochondrial disorders with mtDNA instability

**Figure 5.**
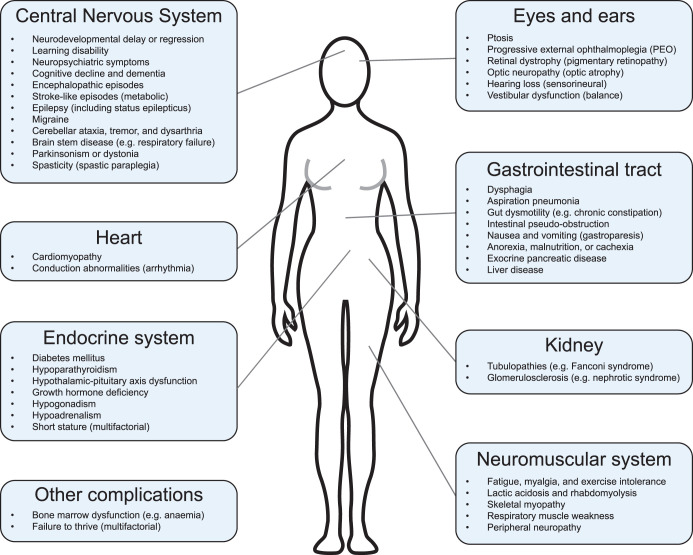
Clinical manifestations of mtDNA instability. Symptoms associated with either single, large-scale mtDNA deletion disorders or mtDNA maintenance disorders are shown that affect the central nervous system [14,231,233–237,309–311], eyes and ears [14,231–238,243,312], heart [14,179,231,233–235,237,287,290–292], gastrointestinal tract [14,231,233–235,237,313], endocrine system [14,231,233–236,314,315], kidney [14,231,233–235,316,317], neuromuscular system [14,231–238,243], as well as other complications [14,231,233–237].

Most mitochondrial disease phenotypes are multisystemic and notoriously heterogeneous, depending upon the gene or variant involved, and mtDNA instability disorders are no exception (Figure 5) [14,231–237,243]. While we aim to describe disorders associated with mtDNA instability, a comprehensive clinical description of these conditions is out of the scope of this review and can be found elsewhere [14,231–238,243].

Skeletal myopathy with weakness, fatigue, and declining mobility and exercise tolerance are common [14,231–238,243]. Eye movements are often restricted due to extra-ocular muscle weakness, termed progressive ophthalmoplegia (PEO), often with reduced ability to raise the upper eyelid (ptosis) [14,231–238,243]. Muscle weakness may affect speech (dysphonia) and swallowing (dysphagia), which may lead to malnutrition and aspiration pneumonia [14,231–238,243].

Nervous system disease is heterogeneous but common, and different neurological structures may contribute, often simultaneously, to similar clinical features such as visual impairment due to retinal, optic nerve and/or brain disease (e.g. stroke-like episodes), or loss of coordination (ataxia) due to cerebellum, spinal cord and/or peripheral nerve disease [14,231–238,243]. Nervous system involvement may be severe, disabling, or fatal, including epilepsy, stroke-like episodes, cognitive impairment, neuropsychiatric symptoms, sensory impairment, hearing loss, and motor disability due to spasticity, dystonia, rigidity and/or ataxia [14,231–238,318]. Moreover, early onset disease may cause failure to thrive, neurodevelopmental delay and intellectual disability [14,231,233–237].

Endocrinological (e.g. diabetes), gastrointestinal, cardiac, renal, and liver disease may also be prominent and potentially life-threatening [14,231–237]. Adult-onset SLSD and MMD tend to cause myopathic phenotypes that progress over decades, while more damaging genotypes cause early-onset, multisystemic, rapidly progressive disease, sometimes with superimposed metabolic crisis events (e.g. Leigh syndrome, Pearson syndrome (PS) and stroke-like episodes) [14,231–238,243].

### Single, large-scale deletions

SLSD comprise a spectrum of overlapping phenotypes, including the classically described PS, Kearns-Sayre syndrome (KSS) and chronic progressive external ophthalmoplegia (CPEO) [231,233–238]. PS is characterised by infantile sideroblastic anaemia, exocrine pancreatic dysfunction, and failure to thrive, as well as renal and liver disease [233–236,239–241,319]. Despite best medical care (e.g. blood transfusions), neutropenia-related infections and severe lactic acidosis may be fatal [234,239–241,319], however survivors often convert into a KSS phenotype [236,239–241]. Classically, KSS manifests before 20 years of age with PEO, ptosis, pigmentary retinopathy, and cardiac disease, but myopathy, ataxia, cognitive impairment, hearing loss, dysphagia, gastrointestinal complications, renal disease, seizures, and endocrinopathies are also part of the KSS spectrum [231,233–236,238,320]. Classically, CPEO is an adult-onset progressive myopathy with PEO, ptosis, proximal limb weakness, poor exercise tolerance, and dysphagia, however, multisystemic features may emerge with age (CPEO plus) [231,233,235,236,238,243,320].

Despite significant genotype-phenotype variability [231,233,234,236,238,321,322], the deletion load in SLSD correlates inversely with age of onset [233,234] and positively with clinical severity [238,322]. Total and wild type numbers of mtDNA copies may also modulate the level of mitochondrial dysfunction in tissues [10,321]. In some studies, but not all, the deletion size showed inverse correlation with age of onset and positive correlation with multisystemic disease, clinical progression, or severity [233,236,238]. In some studies, the position of the deletion and set of lost genes also modulated age of onset, disease severity [236,322] and level of mitochondrial dysfunction [238,323]. Phenotypes are assigned based on presenting features but tend to evolve along the SLSD spectrum over time [233,236,239–241]. Remarkably, the proportion of the deletion in SLSD declines in mitotic tissues (e.g. blood) but rises in post-mitotic tissues (e.g. skeletal muscle) with age [240,305]. For instance, PS presents with high deletion load (> 70%) that declines rapidly with age in the liver and blood of a PS mouse model, and in blood of patients, as they recover haematopoietic function [240]. Simultaneously, SLSD clonally expand to high levels in the skeletal muscle of KSS patients [305], as well as in muscle of PS mice and PS survivors who convert into a KSS phenotype [240].

### mtDNA maintenance disorders

MMD represent a wide range of autosomal dominant or recessive disorders directly impairing mtDNA replication (*POLG*, *POLG2*, *TWNK*, *TFAM*, *RNASEH1*, *MGME1*, *DNA2* and *TOP3A*), disrupting mitochondrial nucleotide metabolism (*TK2*, *DGUOK*, *SUCLG1*, *SUCLA2*, *ABAT*, *RRM2B*, *TYMP*, *SLC25A4*, *AGK*, and *MPV17*), or affecting mitochondrial dynamics (*OPA1*, *MFN2*, and *FBXL4*) [9,14,16,232,235,237,243,324,325]. More deleterious and often recessive variants tend to cause mtDNA depletion leading to early onset, severe, multisystemic and potentially fatal conditions, while less severe and often dominant variants cause more slowly progressive and often myopathic adult-onset diseases with accumulation of multiple mtDNA deletions [14,16,17,232,235,243,306,308]. Overall, myopathy is common, with variable progression and anatomical patterns, from isolated PEO, often with ptosis, to PEO with severe axial (core) and proximal (limb girdle) muscle weakness, sometimes with severe dysphagia and respiratory insufficiency [14,16,232,235,243]. The PEO phenotype, which has PEO as its defining feature, can include almost any other skeletal muscle involvement, with ptosis and proximal myopathy as common features, especially if considering its natural history of disease progression [14,16,232,235,243].

Depending on genotype and age of onset, additional neurological features may be present, including epilepsy, stroke-like episodes, parkinsonism, ataxia, neuropathy, and optic atrophy [14,16,232,235,237,244–247]. These may be prominent, often in various combinations (e.g. *POLG* variants) [244–247], or may be the main or single manifestation (e.g. optic atrophy in *OPA1* variants) [14,16,232,235,293]. Non-neurological complications may also be present and prominent in some genotypes, such as severe and potentially life-threatening gastrointestinal disease (e.g. *TYMP*, *POLG*, *RRM2B* and *MGME1* variants) [90,244,246,247,268–272], liver disease (e.g. *POLG*, *MPV17*, *DGUOK* and *TFAM* variants) [16,232,244,246–249,267,276–278] and cardiac disease (e.g. *AGK*, *SLC25A4* and *TOP3A* variants) [179,287,290–292].

### Disorders of mtDNA replication

Variants of *TWNK* and *POLG* collectively cause disease in 1.0 in 100 000 adults in the North East of England [242] and, in other cohort studies were found to explain 16% of all cases exhibiting PEO with or without other clinical features, 16% of cases of PEO phenotype without other features [243], and 41% of cases of autosomal PEO phenotype [250]. In the North East of England, dominant *TWNK* variants were the most common cause of mtDNA replication disease, affecting 0.7 per 100 000 adults [242], and typically cause adult-onset PEO, ptosis, and proximal weakness [79,326]. PEO may be found in 86% of *TWNK* cases, with 96% being classified as ‘pure’ PEO [243], while *TWNK* variants may explain 6% of all cases with PEO [243] and 16.5% of autosomal PEO cases [250]. More rarely, recessive *TWNK* variants cause mtDNA depletion and severe multisystemic neurodegenerative disorders, with liver disease, untreatable epilepsy, and infantile death [327–329].

Recessive *POLG* disease affects 0.3 per 100 000 adults in the North East of England [242], however, *POLG* disease was found to be the most prevalent monogenic mitochondrial disease (10%) in an Australian adult cohort [251]. *POLG* disease is a spectrum of overlapping features emerging from early infancy to late adulthood and punctuated by classically defined syndromes [244]. Recessive variants, often causing mtDNA depletion, manifest early in life as multisystemic and rapidly progressive neurodegeneration, often with refractory epilepsy, and end-stage liver failure, including myocerebrohepatopathy spectrum diseases and Alpers-Huttenlocher syndrome [244–249]. Less deleterious variants may manifest before adulthood as multisystemic and neurodegenerative phenotypes including the classical myoclonic epilepsy, myopathy and sensory ataxia (MEMSA), spinocerebellar ataxia with epilepsy (SCAE), mitochondrial recessive ataxia syndrome (MIRAS) and sensory ataxia, neuropathy, dysarthria and ophthalmoplegia (SANDO) syndromes [244–249]. Dominant *POLG* variants often manifest as adult-onset slowly progressive disease, usually PEO, ptosis, and myopathy, although other features may manifest later in life [244,252]. Nevertheless, PEO may be found in 83% of *POLG* cases, with 47% being classified as ‘pure’ PEO [243], while *POLG* variants may explain 10% of all cases with PEO [243] and 25% of autosomal cases with PEO [250].

Amongst other mtDNA replication factors that have been associated with mitochondrial disease, dominant *POLG2* variants cause PEO, ptosis, and myopathy, from infancy to late adulthood, often with additional neurological and non-neurological features [253–255], while recessive variants cause severe phenotypes akin to the *POLG* spectrum [255,256]. A *TFAM* variant has been reported to cause neonatal liver failure with mtDNA depletion in two consanguineous siblings of Colombian-Basque descent [267]. Recessive *TOP3A* variants either cause mtDNA depletion and severe multisystemic neonatal disease, or adult-onset PEO, ptosis, and proximal myopathy, with peripheral neuropathy and cardiac disease, in association with multiple mtDNA deletions [179,259,260]. The spectrum of *TOP3A* disease is complicated by the presence of mitochondrial and nuclear isoforms, with more severe variants causing deficiency of the nuclear isoform and leading to a Bloom syndrome-like disorder characterised by short stature and predisposition to tumour formation [179,261,262].

Autosomal variants affecting nucleases that support mtDNA replication, including *RNASEH1* [250,257,258], *MGME1* [90,263], and *DNA2* [264–266], can also cause mtDNA replication disorders, often with prominent and marked myopathy, including respiratory weakness, and a spectrum of neurological and non-neurological features, including gastrointestinal disease [16,232]. Recessive *RNASEH1* variants cause adult-onset PEO, ptosis, myopathy, respiratory weakness, dysphagia, cerebellar disease, and neuropathy, with multiple mtDNA deletions [250,257,258]. Recessive *MGME1* variants cause PEO, ptosis, and marked myopathy with respiratory weakness in childhood or early adulthood, with mtDNA depletion or multiple deletions [90,263], and may cause severe emaciation due to gastrointestinal disease [90]. Dominant *DNA2* variants cause childhood to adulthood onset of slowly progressive PEO, ptosis, and myopathy, with multiple mtDNA deletions [264]. However, truncating and frameshift variants may cause early onset myopathy [266] with rhabdomyolysis and cardiac disease [265].

### MMD of deoxynucleotide triphosphate metabolism and mitochondrial dynamics

In addition to disorders of mtDNA replication, deletions in mtDNA have also been observed in cases of mitochondrial disease associated with pathogenic variants in factors required for the availability of mitochondrial deoxynucleotide triphosphates (dNTPs), as well as in proteins involved in mitochondrial dynamics.

Variants affecting the availability or balance of mitochondrial dNTPs may cause MMD with a wide range of multisystemic features, disease severity and age of onset [16,232,237,248,249,276,307,330–332]. This may involve proteins required for mitochondrial dNTP salvage, for cytosolic *de novo* dNTP synthesis, or for the transport of nucleotide precursors across mitochondrial membranes. Variants in *TK2* and *DGUOK* disrupt mitochondrial pyrimidine and purine salvage pathways, respectively [16,232,330,332]. Both succinyl-CoA ligase (SUCL) and ABAT are required to convert deoxyadenosine (ADP) or guanosine (GDP) diphosphates to their triphosphorylated forms [232,284]. Variants in *SUCLA2* [281] and *SUCLG1* [283] (subunits of SUCL [282]), and in *ABAT* [284], cause mtDNA depletion syndromes associated with disrupted mitochondrial dNTP pools [232]. Mitochondrial nucleotide pools further rely on *de novo* synthesis in the cytosol by ribonucleotide reductase, and may be indirectly perturbed by variants in its regulatory RRM2B subunit [268], known to cause both mtDNA depletion and multiple mtDNA deletions [232,268,269]. Finally, *TYMP* variants may impair thymidine nucleotide metabolism, with the accumulation of pyrimidine nucleosides that disrupt mtDNA synthesis [271,272]. Cytosolic dNTPs must be imported into the matrix, and variants in related IMM proteins have been found to cause multiple mtDNA deletions and, more frequently, mtDNA depletion syndromes [249,276,307,330,331]. For instance, acylglycerol kinase (AGK) maintains an ideal IMM composition [290–292] for efficient insertion of the ADP/ATP translocator SLC25A4, which is critical to maintain the mitochondrial dNTP pool and metabolic balance [288]. MPV17 is another IMM protein which is required to import deoxythymidine monophosphate and sustain mtDNA synthesis [333]. Variants in *AGK* [290–292], *SLC25A4* [288] or *MPV17* [248,249,276,277] have all been reported to cause MMD with mtDNA depletion or multiple deletions.

Most recessive *DGUOK* and *MPV17* variants cause severe and often fatal nervous system and liver disease before adulthood due to mtDNA depletion [248,249,276–278]. However, less damaging variants may cause mtDNA deletions, and lead to adult-onset PEO, ptosis, and myopathy in the case of *DGUOK* [279,280] and myopathy with neuropathy in the case of *MPV17* [277]. Recessive *TK2* variants cause mtDNA depletion with early onset severe myopathy with respiratory failure [273], but intermediate phenotypes and less severe adult-onset myopathic forms with mtDNA multiple deletions have also been described [274,275]. Recessive *SUCLA2* [281,282] or *SUCLG1* [282,283] variants cause multisystemic infantile mtDNA depletion with severe muscle and central nervous disease (encephalomyopathic syndrome), often with additional non-neurological features, leading to death during childhood [248,282]. Recessive variants in *ABAT* can cause early onset, severe and fatal encephalopathy, with documented mtDNA depletion in patients [284]. However, because ABAT is also required for the catabolism of the inhibitory neurotransmitter gamma-aminobutyric acid (GABA) [285], the phenotype has so far been mainly linked to GABA accumulation [286]. Recessive *TYMP* variants classically cause a progressive and multisystemic syndrome with potentially fatal gastrointestinal dysmotility and cachexia, combined with myopathy and neuropathy, named mitochondrial neurogastrointestinal encephalopathy (MNGIE), with both depletion and multiple mtDNA deletions having been described [270–272]. However, less damaging variants cause less severe and often late-onset disease [270]. Curiously, recessive *RRM2B* variants often cause even more severe, rapidly progressive and multisystemic mtDNA depletion syndromes with severe myopathy, gastrointestinal, nervous system (MNGIE-type), and sometimes renal disease, which are often fatal within infancy [268,269]. A less severe PEO, ptosis, and myopathy phenotype, manifesting before early adulthood, is associated with dominant *RRM2B* variants causing mtDNA multiple deletions, with less frequent or severe multisystemic features [269]. *AGK* [290,292] and *SLC25A4* [287–289] variants often cause cardiac and skeletal myopathy frequently with mtDNA depletion [330]. Surprisingly, dominant *SLC25A4* disease can manifest either at birth, with respiratory failure, feeding difficulties and mtDNA depletion [287], or as adult-onset slowly progressive PEO, ptosis, and myopathy with multiple mtDNA deletions [287,289]. Furthermore, recessive *AGK* variants cause potentially fatal infantile respiratory and cardiac failure [290–292], but recessive *SLC25A4* variants cause a childhood-onset intermediate form of cardiac and skeletal myopathy [287].

Mitochondrial dynamics, particularly mitochondrial fusion, is also required to maintain mtDNA stability and is disrupted by variants in *OPA1* [293], *MFN2* [296,297,334] and *FBXL4* [302,303]. As described above in relation to variants in the mtDNA replication machinery, more severe variants are associated with mtDNA depletion in childhood, whereas less severe variants may cause the accumulation of multiple mtDNA deletions in individuals with adult-onset mitochondrial disease. *OPA1* variants mostly cause autosomal dominant optic atrophy with incomplete penetrance, starting in early childhood or early adulthood (bimodal), occasionally with additional features including myopathy, neuropathy, hearing loss, spasticity, and cerebellar disease [293,294]. Deletions within mtDNA have been observed associated with multisystemic OPA1 disease in adults [293,335]. However, compound heterozygosity may also cause mtDNA depletion, manifesting at birth with marked cardiac, myopathic and nervous system disease causing early death [295]. Although phenotypically variable, *MFN2*-related disease preferentially damages the peripheral nervous system, causing dominant or recessive motor and sensory neuropathic phenotypes, with variable additional features, most notably optic atrophy, and multiple mtDNA deletions [296–301]. Finally, recessive *FBXL4* variants cause a severe, multisystemic and often fatal, infantile encephalomyopathic syndrome associated with mtDNA depletion [302–304].

## Clonal expansion and tissue specificity of mtDNA rearrangements

### Mechanism of clonal expansion

mtDNA mutations have been observed to accumulate over the course of human lifespan, and contribute to the pathogenesis of ageing and disease [336]. Several theories have been proposed to explain the mechanism by which mtDNA mutations accumulate; a process called clonal expansion. Increasingly, it seems apparent that no single mechanism is consistent with all mtDNA mutations and cell types. Two prominent early theories of clonal expansion are referred to as ‘survival of the smallest’ [337] and ‘survival of the sickest’ [338], although both theories have largely fallen out of favour. The ‘survival of the sickest’ theory posited that mitochondria containing a mutation-carrying mtDNA molecule will be less efficient at OXPHOS and will therefore produce less ROS, and as such will go undetected by quality control mechanisms designed to remove damaged mitochondria [338]. The ‘survival of the smallest’ theory alternatively suggested that an mtDNA molecule containing a deletion will take less time to replicate than a wild-type molecule because of its smaller size, and will therefore out-compete the wild type mtDNA over time [337]. This hypothesis was supported by the observation that, when mtDNA copy number is artificially depleted, the rapid mtDNA replication that repopulates mtDNA copy number leads to an increase in the proportion of the deletion-containing mtDNA species [339]. However, data from *C. elegans* that has studied two different sizes of mtDNA deletion has suggested that deletion size is not an important factor in clonal expansion [340]. Furthermore, deletion size does not seem to have an impact in human skeletal muscle, as deletions of different sizes appear to have no impact on the size of the respiratory chain deficient region observed in a muscle fibre [308], and so it was concluded that mtDNA deletion size does not impact the rate at which an mtDNA deletion clonally expands. It therefore seems more likely that mtDNA size is only important under conditions of relaxed replication in order to replenish mtDNA copy number and is not rate limiting in human tissues. Theories of clonal expansion based upon the size of the molecule would also be unable to explain how mtDNA point mutations clonally expand, as mutation-containing molecules are the same size as wild type mtDNA.

There is now consensus that the clonal expansion of mtDNA point mutations can be explained by random genetic drift [341]. Random drift suggests that mtDNA replication under relaxed copy number control is sufficient to cause a stochastic accumulation of mtDNA point mutations over time [342]. As yet it is unclear whether random genetic drift could also explain the clonal expansion of mtDNA deletions, and as such further theories have been suggested to explain their accumulation.

One such theory suggests a feedback loop coordinating mtDNA translation and replication, whereby a reduced rate of translation of specific mtDNA-encoded proteins, which could be lost due to a deletion, would be linked to an increased rate of mtDNA replication in order to allow more protein synthesis [343,344]. Whilst this is an interesting theory, an assessment of the mtDNA deletion spectra reported in MitoBreak [168] does not indicate that specific genes are consistently removed in all of the reported, clonally expanded mtDNA deletions.

Another theory has approached this question with a focus on the tissue-specific environment, as opposed to mitochondrial genetics and biology. Based on the smallest regions of mitochondrial dysfunction in skeletal muscle being found consistently adjacent to nuclei, it was suggested that the myonuclei in skeletal muscle offer a replicative advantage to mtDNA deletions in perinuclear mitochondria, because their close proximity to the nucleus facilitates rapid mito-nuclear signalling and an increased rate of mtDNA replication [306]. Whilst the data reported supports the impact of this proximity, there are still unanswered questions around the exact role of mito-nuclear signalling and mtDNA replication in the clonal expansion of mtDNA deletions in skeletal muscle, in particular whether the rate of mtDNA replication is constitutively higher in the perinuclear region or is triggered by mitochondrial dysfunction and retrograde signalling. *In silico* modelling of a spatial unit of mtDNA dynamics in a single sarcomere of skeletal muscle has demonstrated that stochasticity, density of mutated mtDNA and a spatial structure are sufficient for clonal expansion; this theory was termed ‘stochastic survival of the densest’ [345]. This suggestion is similar to the previously-mentioned perinuclear niche hypothesis in that it considers highly structured skeletal muscle, although the modelling focuses on only a single sarcomere. Both studies focus on the unique tissue architecture of skeletal muscle, with multiple nuclei and a densely packed cytoskeletal structure that restricts mitochondrial transport, meaning that this phenomenon may not occur in other cell types.

### Tissue specificity of clonal expansion

An interesting difference in the literature between mtDNA point mutations and mtDNA deletions is that mtDNA deletions appear to clonally expand preferentially to mtDNA point mutations in post-mitotic tissues. In comparison, mtDNA point mutations are more commonly seen in mitotic cells [215]. A distinct caveat of this observation is that there are only few studies that have systematically investigated clonally expanded mtDNA point mutations in post-mitotic tissues using unbiased methods [346]. This difference between mitotic and post-mitotic tissues could be explained by mtDNA deletions being more damaging, and therefore less likely to be propagated in cells that must divide regularly. However, it is also possible that the prior assumption that mtDNA deletions will be observed in post-mitotic cells, and that mtDNA point mutations will be observed in mitotic cells, biases the types of investigations that are carried out and, as a result, the currently available data is incomplete. Differences in inheritance patterns between mtDNA deletions and point mutations must also be taken into account. For example, cells with cytochrome c oxidase (COX) deficiency can be observed in patients with inherited m.8344A > G mutations, however, it is difficult to determine whether these mtDNA mutations have clonally expanded or merely persisted due to their inherited nature. Investigations of sporadic, rather than inherited, mtDNA point mutations would be useful to confidently determine whether mtDNA point mutations do clonally expand in skeletal muscle.

Differences in deletion spectra and levels have been observed between different diseases [168,347], with deletions within both the major and minor arc observed in MMD, inclusion body myositis, ageing, and tumours, but typically restricted to the major arc in Parkinson's disease. In brain, the proportion of deletion-containing mtDNA molecules in single neurons from the substantia nigra varies between 20% and 70%, associated with either Parkinson's or healthy ageing [348]. In skeletal muscle, proportions of deletion-containing molecules as high as 99% have been detected in MMD and ageing [17,164]. This may relate to the ability of skeletal muscle to buffer high levels of mtDNA deletions using adjacent healthy mitochondria from the same cell [164] and a higher sensitivity of neurons to mitochondrial dysfunction, leading to neuronal cell death at higher deletion loads.

## Conclusions

The study of mtDNA replication and instability has long been challenging, because of the multi-copy nature of mtDNA, differences in opinion over the mechanism of mtDNA replication, the lack of suitable systems to test models of deletion formation and accumulation, and the complex tissue specificity and different inheritance patterns of mitochondrial disorders.

Data supports the idea that deletions can be formed either due to aberrant mtDNA replication or following mtDNA breakage, although the relative contributions of these processes to the spectrum of mtDNA deletions seen in individual mitochondrial diseases and ageing is yet to be determined. The existence and functional significance of mtDNA duplications, seen associated with the loss of specific protein activities, also remains relatively unexplored and will be an interesting topic of future study.

Understanding the complex tissue specificity of mitochondrial diseases remains one of the greatest outstanding problems in the field, of which the tissue specificity of SLSDs and MMDs are a contributing factor. Studies in cultured cells, as well as detailed *in vitro* work, has allowed for the development of detailed molecular mechanisms for mtDNA replication and the formation of deletions. In the future, a better understanding of how the rate of mtDNA replication and mtDNA damage differs between tissues will be required to better establish where and why mtDNA mutations and deletions accumulate. This may also help to answer the longstanding question of why mtDNA point mutations apparently predominate in mitotic cells, while mtDNA deletions are primarily found in post-mitotic cells.

## Open Access

Open access for this article was enabled by the participation of Newcastle University in an all-inclusive *Read & Publish* agreement with Portland Press and the Biochemical Society under a transformative agreement with JISC.

## Abbreviations

2D-AGE: 2-dimensional agarose gel electrophoresis
AGK: acylglycerol kinase
CPEO: chronic progressive external ophthalmoplegia
CSB: Conserved Sequence Box
dNTP: deoxynucleotide triphosphate
GABA: gamma-aminobutyric acid
HR: homologous recombination
HSP: heavy-strand promoter
IMM: inner mitochondrial membrane
KSS: Kearns-Sayre syndrome
LSP: light-strand promoter
MEMSA: myoclonic epilepsy, myopathy and sensory ataxia
MIRAS: mitochondrial recessive ataxia syndrome
MMD: mitochondrial DNA maintenance disorders
MNGIE: mitochondrial neurogastrointestinal encephalopathy
mtDNA: mitochondrial DNA
NCR: non-coding region
OXPHOS: oxidative phosphorylation
PEO: progressive ophthalmoplegia
PS: Pearson syndrome
SDM: strand displacement model
SLSD: single, large-scale deletions
ssDNA: single-stranded DNA
TAS: termination-associated sequence
TEM: transmission electron microscopy

## References

[1] Gray, M.W., Burger, G. and Lang, B.F. (1999) Mitochondrial evolution. Science 283, 1476–1481 10.1126/science.283.5407.1476 10066161

[2] Nass, M.M. and Nass, S. (1963) Intramitochondrial fibers with DNA characteristics. I. Fixation and electron staining reactions. J. Cell Biol. 19, 593–611 10.1083/jcb.19.3.593 14086138 PMC2106331

[3] Anderson, S., Bankier, A.T., Barrell, B.G., de Bruijn, M.H., Coulson, A.R., Drouin, J. et al. (1981) Sequence and organization of the human mitochondrial genome. Nature 290, 457–465 10.1038/290457a0 7219534

[4] Andrews, R.M., Kubacka, I., Chinnery, P.F., Lightowlers, R.N., Turnbull, D.M. and Howell, N. (1999) Reanalysis and revision of the Cambridge reference sequence for human mitochondrial DNA. Nat. Genet. 23, 147 10.1038/13779 10508508

[5] Antes, A., Tappin, I., Chung, S., Lim, R., Lu, B., Parrott, A.M. et al. (2010) Differential regulation of full-length genome and a single-stranded 7S DNA along the cell cycle in human mitochondria. Nucleic Acids Res. 38, 6466–6476 10.1093/nar/gkq493 20530535 PMC2965228

[6] Gorman, G.S., Chinnery, P.F., DiMauro, S., Hirano, M., Koga, Y., McFarland, R. et al. (2016) Mitochondrial diseases. Nat. Rev. Dis. Primers. 2, 16080 10.1038/nrdp.2016.80 27775730

[7] Wallace, D.C. and Chalkia, D. (2013) Mitochondrial DNA genetics and the heteroplasmy conundrum in evolution and disease. Cold Spring Harb. Perspect. Biol. 5, a021220 10.1101/cshperspect.a021220 24186072 PMC3809581

[8] Stewart, J.B. and Chinnery, P.F. (2015) The dynamics of mitochondrial DNA heteroplasmy: implications for human health and disease. Nat. Rev. Genet. 16, 530–542 10.1038/nrg3966 26281784

[9] Alston, C.L., Rocha, M.C., Lax, N.Z., Turnbull, D.M. and Taylor, R.W. (2017) The genetics and pathology of mitochondrial disease. J. Pathol. 241, 236–250 10.1002/path.4809 27659608 PMC5215404

[10] Durham, S.E., Bonilla, E., Samuels, D.C., DiMauro, S. and Chinnery, P.F. (2005) Mitochondrial DNA copy number threshold in mtDNA depletion myopathy. Neurology 65, 453–455 10.1212/01.wnl.0000171861.30277.88 16087914

[11] Shoffner, J.M., Lott, M.T., Lezza, A.M., Seibel, P., Ballinger, S.W. and Wallace, D.C. (1990) Myoclonic epilepsy and ragged-red fiber disease (MERRF) is associated with a mitochondrial DNA tRNA(Lys) mutation. Cell 61, 931–937 10.1016/0092-8674(90)90059-N 2112427

[12] Boulet, L., Karpati, G. and Shoubridge, E.A. (1992) Distribution and threshold expression of the tRNA(Lys) mutation in skeletal muscle of patients with myoclonic epilepsy and ragged-red fibers (MERRF). Am. J. Hum. Genet. 51, 1187–1200 1334369 PMC1682926

[13] Rossignol, R., Faustin, B., Rocher, C., Malgat, M., Mazat, J.P. and Letellier, T. (2003) Mitochondrial threshold effects. Biochem. J. 370, 751–762 10.1042/bj20021594 12467494 PMC1223225

[14] Viscomi, C. and Zeviani, M. (2017) MtDNA-maintenance defects: syndromes and genes. J. Inherit. Metab. Dis. 40, 587–599 10.1007/s10545-017-0027-5 28324239 PMC5500664

[15] Chapman, J., Ng, Y.S. and Nicholls, T.J. (2020) The maintenance of mitochondrial DNA integrity and dynamics by mitochondrial membranes. Life (Basel) 10, 164 10.3390/life10090164 32858900 PMC7555930

[16] El-Hattab, A.W., Craigen, W.J. and Scaglia, F. (2017) Mitochondrial DNA maintenance defects. Biochim. Biophys. Acta Mol. Basis Dis. 1863, 1539–1555 10.1016/j.bbadis.2017.02.017 28215579

[17] Lehmann, D., Tuppen, H.A.L., Campbell, G.E., Alston, C.L., Lawless, C., Rosa, H.S. et al. (2019) Understanding mitochondrial DNA maintenance disorders at the single muscle fibre level. Nucleic Acids Res. 47, 7430–7443 10.1093/nar/gkz472 31147703 PMC6698645

[18] Richter-Dennerlein, R., Oeljeklaus, S., Lorenzi, I., Ronsör, C., Bareth, B., Schendzielorz, A.B. et al. (2016) Mitochondrial protein synthesis adapts to influx of nuclear-encoded protein. Cell 167, 471–483.e410 10.1016/j.cell.2016.09.003 27693358 PMC5055049

[19] Zorkau, M., Albus, C.A., Berlinguer-Palmini, R., Chrzanowska-Lightowlers, Z.M.A. and Lightowlers, R.N. (2021) High-resolution imaging reveals compartmentalization of mitochondrial protein synthesis in cultured human cells. Proc. Natl Acad. Sci. U.S.A. 118, e2008778118 10.1073/pnas.2008778118 33526660 PMC8017971

[20] Itoh, Y., Andréll, J., Choi, A., Richter, U., Maiti, P., Best, R.B. et al. (2021) Mechanism of membrane-tethered mitochondrial protein synthesis. Science 371, 846–849 10.1126/science.abe0763 33602856 PMC7610362

[21] Gustafsson, C.M., Falkenberg, M. and Larsson, N.G. (2016) Maintenance and expression of mammalian mitochondrial DNA. Annu. Rev. Biochem. 85, 133–160 10.1146/annurev-biochem-060815-014402 27023847

[22] Montoya, J., Christianson, T., Levens, D., Rabinowitz, M. and Attardi, G. (1982) Identification of initiation sites for heavy-strand and light-strand transcription in human mitochondrial DNA. Proc. Natl Acad. Sci. U.S.A. 79, 7195–7199 10.1073/pnas.79.23.7195 6185947 PMC347305

[23] Holzmann, J., Frank, P., Löffler, E., Bennett, K.L., Gerner, C. and Rossmanith, W. (2008) RNase P without RNA: identification and functional reconstitution of the human mitochondrial tRNA processing enzyme. Cell 135, 462–474 10.1016/j.cell.2008.09.013 18984158

[24] Sanchez, M.I., Mercer, T.R., Davies, S.M., Shearwood, A.M., Nygård, K.K., Richman, T.R. et al. (2011) RNA processing in human mitochondria. Cell Cycle 10, 2904–2916 10.4161/cc.10.17.17060 21857155

[25] Brzezniak, L.K., Bijata, M., Szczesny, R.J. and Stepien, P.P. (2011) Involvement of human ELAC2 gene product in 3′ end processing of mitochondrial tRNAs. RNA Biol. 8, 616–626 10.4161/rna.8.4.15393 21593607

[26] Ojala, D., Montoya, J. and Attardi, G. (1981) tRNA punctuation model of RNA processing in human mitochondria. Nature 290, 470–474 10.1038/290470a0 7219536

[27] Litonin, D., Sologub, M., Shi, Y., Savkina, M., Anikin, M., Falkenberg, M. et al. (2010) Human mitochondrial transcription revisited: only TFAM and TFB2M are required for transcription of the mitochondrial genes in vitro. J. Biol. Chem. 285, 18129–18133 10.1074/jbc.C110.128918 20410300 PMC2881736

[28] Zollo, O., Tiranti, V. and Sondheimer, N. (2012) Transcriptional requirements of the distal heavy-strand promoter of mtDNA. Proc. Natl Acad. Sci. U.S.A. 109, 6508–6512 10.1073/pnas.1118594109 22454497 PMC3340101

[29] Tan, B.G., Gustafsson, C.M. and Falkenberg, M. (2023) Mechanisms and regulation of human mitochondrial transcription. Nat. Rev. Mol. Cell Biol. 25, 119–132 10.1038/s41580-023-00661-4 37783784

[30] Yan, B., Tzertzinis, G., Schildkraut, I. and Ettwiller, L. (2022) Comprehensive determination of transcription start sites derived from all RNA polymerases using ReCappable-seq. Genome Res. 32, 162–174 10.1101/gr.275784.121 34815308 PMC8744680

[31] Tan, B.G., Mutti, C.D., Shi, Y., Xie, X., Zhu, X., Silva-Pinheiro, P. et al. (2022) The human mitochondrial genome contains a second light strand promoter. Mol. Cell 82, 3646–3660.e3649 10.1016/j.molcel.2022.08.011 36044900 PMC7619432

[32] Jiang, M., Xie, X., Zhu, X., Jiang, S., Milenkovic, D., Misic, J. et al. (2021) The mitochondrial single-stranded DNA binding protein is essential for initiation of mtDNA replication. Sci. Adv. 7, eabf8631 10.1126/sciadv.abf8631 34215584 PMC11057760

[33] Chang, D.D. and Clayton, D.A. (1985) Priming of human mitochondrial DNA replication occurs at the light-strand promoter. Proc. Natl Acad. Sci. U.S.A. 82, 351–355 10.1073/pnas.82.2.351 2982153 PMC397036

[34] Martens, P.A. and Clayton, D.A. (1979) Mechanism of mitochondrial DNA replication in mouse L-cells: localization and sequence of the light-strand origin of replication. J. Mol. Biol. 135, 327–351 10.1016/0022-2836(79)90440-6 537081

[35] Kasamatsu, H., Robberson, D.L. and Vinograd, J. (1971) A novel closed-circular mitochondrial DNA with properties of a replicating intermediate. Proc. Natl Acad. Sci. U.S.A. 68, 2252–2257 10.1073/pnas.68.9.2252 5289384 PMC389395

[36] Tapper, D.P. and Clayton, D.A. (1981) Mechanism of replication of human mitochondrial DNA. Localization of the 5′ ends of nascent daughter strands. J. Biol. Chem. 256, 5109–5115 10.1016/S0021-9258(19)69373-7 6262317

[37] Nicholls, T.J. and Minczuk, M. (2014) In D-loop: 40 years of mitochondrial 7S DNA. Exp. Gerontol. 56, 175–181 10.1016/j.exger.2014.03.027 24709344

[38] Falkenberg, M., Gaspari, M., Rantanen, A., Trifunovic, A., Larsson, N.G. and Gustafsson, C.M. (2002) Mitochondrial transcription factors B1 and B2 activate transcription of human mtDNA. Nat. Genet. 31, 289–294 10.1038/ng909 12068295

[39] Shi, Y.H., Dierckx, A., Wanrooij, P.H., Wanrooij, S., Larsson, N.G., Wilhelmsson, L.M. et al. (2012) Mammalian transcription factor A is a core component of the mitochondrial transcription machinery. Proc. Natl Acad. Sci. U.S.A. 109, 16510–16515 10.1073/pnas.1119738109 23012404 PMC3478657

[40] Greenleaf, A.L., Kelly, J.L. and Lehman, I.R. (1986) Yeast Rpo41 gene-product is required for transcription and maintenance of the mitochondrial genome. Proc. Natl Acad. Sci. U.S.A. 83, 3391–3394 10.1073/pnas.83.10.3391 3517858 PMC323519

[41] Masters, B.S., Stohl, L.L. and Clayton, D.A. (1987) Yeast mitochondrial RNA-polymerase is homologous to those encoded by bacteriophage-T3 and bacteriophage-T7. Cell 51, 89–99 10.1016/0092-8674(87)90013-4 3308116

[42] Tiranti, V., Savoia, A., Forti, F., DApolito, M.F., Centra, M., Racchi, M. et al. (1997) Identification of the gene encoding the human mitochondrial RNA polymerase (h-mtRPOL) by cyberscreening of the expressed sequence tags database. Hum. Mol. Genet. 6, 615–625 10.1093/hmg/6.4.615 9097968

[43] Gaspari, M., Falkenberg, M., Larsson, N.G. and Gustafsson, C.M. (2004) The mitochondrial RNA polymerase contributes critically to promoter specificity in mammalian cells. EMBO J. 23, 4606–4614 10.1038/sj.emboj.7600465 15526033 PMC533051

[44] Hillen, H.S., Morozov, Y.I., Sarfallah, A., Temiakov, D. and Cramer, P. (2017) Structural basis of mitochondrial transcription initiation. Cell 171, 1072 10.1016/j.cell.2017.10.036 29149603 PMC6590061

[45] Seidel-Rogol, B.L., McCulloch, V. and Shadel, G.S. (2003) Human mitochondrial transcription factor B1 methylates ribosomal RNA at a conserved stem-loop. Nat. Genet. 33, 23–24 10.1038/ng1064 12496758

[46] Metodiev, M.D., Lesko, N., Park, C.B., Cámara, Y., Shi, Y.H., Wibom, R. et al. (2009) Methylation of 12S rRNA is necessary for in vivo stability of the small subunit of the mammalian mitochondrial ribosome. Cell Metab. 9, 386–397 10.1016/j.cmet.2009.03.001 19356719

[47] Posse, V. and Gustafsson, C.M. (2017) Human mitochondrial transcription factor B2 is required for promoter melting during initiation of transcription. J. Biol. Chem. 292, 2637–2645 10.1074/jbc.M116.751008 28028173 PMC5314162

[48] Morozov, Y.I., Agaronyan, K., Cheung, A.C.M., Anikin, M., Cramer, P. and Temiakov, D. (2014) A novel intermediate in transcription initiation by human mitochondrial RNA polymerase. Nucleic Acids Res. 42, 3884–3893 10.1093/nar/gkt1356 24393772 PMC3973326

[49] Cerritelli, S.M. and Crouch, R.J. (2009) Ribonuclease H: the enzymes in eukaryotes. FEBS J. 276, 1494–1505 10.1111/j.1742-4658.2009.06908.x 19228196 PMC2746905

[50] Posse, V., Al-Behadili, A., Uhler, J.P., Clausen, A.R., Reyes, A., Zeviani, M. et al. (2019) RNase H1 directs origin-specific initiation of DNA replication in human mitochondria. PLoS Genet. 15, e1007781 10.1371/journal.pgen.1007781 30605451 PMC6317783

[51] Holmes, J.B., Akman, G., Wood, S.R., Sakhuja, K., Cerritelli, S.M., Moss, C. et al. (2015) Primer retention owing to the absence of RNase H1 is catastrophic for mitochondrial DNA replication. Proc. Natl Acad. Sci. U.S.A. 112, 9334–9339 10.1073/pnas.1503653112 26162680 PMC4522766

[52] Misic, J., Milenkovic, D., Al-Behadili, A., Xie, X., Jiang, M., Jiang, S. et al. (2022) Mammalian RNase H1 directs RNA primer formation for mtDNA replication initiation and is also necessary for mtDNA replication completion. Nucleic Acids Res. 50, 8749–8766 10.1093/nar/gkac661 35947649 PMC9410905

[53] Suzuki, Y., Holmes, J.B., Cerritelli, S.M., Sakhuja, K., Minczuk, M., Holt, I.J. et al. (2010) An upstream open Reading frame and the context of the two AUG codons affect the abundance of mitochondrial and nuclear RNase H1. Mol. Cell. Biol. 30, 5123–5134 10.1128/MCB.00619-10 20823270 PMC2953059

[54] Cerritelli, S.M., Frolova, E.G. Feng, C., Grinberg, A., Love, P.E. and Crouch, R.J. (2003) Failure to produce mitochondrial DNA results in embryonic lethality in Rnaseh1 null mice. Mol. Cell 11, 807–815 10.1016/S1097-2765(03)00088-1 12667461

[55] Korhonen, J.A., Pham, X.H., Pellegrini, M. and Falkenberg, M. (2004) Reconstitution of a minimal mtDNA replisome. EMBO J. 23, 2423–2429 10.1038/sj.emboj.7600257 15167897 PMC423294

[56] Hance, N., Ekstrand, M.I. and Trifunovic, A. (2005) Mitochondrial DNA polymerase gamma is essential for mammalian embryogenesis. Hum. Mol. Genet. 14, 1775–1783 10.1093/hmg/ddi184 15888483

[57] Humble, M.M., Young, M.J., Foley, J.F., Pandiri, A.R., Travlos, G.S. and Copeland, W.C. (2013) Is essential for mammalian embryogenesis and is required for mtDNA maintenance. Hum. Mol. Genet. 22, 1017–1025 10.1093/hmg/dds506 23197651 PMC3561914

[58] Milenkovic, D., Matic, S., Kühl, I., Ruzzenente, B., Freyer, C., Jemt, E. et al. (2013) TWINKLE is an essential mitochondrial helicase required for synthesis of nascent D-loop strands and complete mtDNA replication. Hum. Mol. Genet. 22, 1983–1993 10.1093/hmg/ddt051 23393161 PMC3633371

[59] Fridlender, B., Fry, M., Weissbach, A. and Bolden, A. (1972) New synthetic RNA-dependent DNA polymerase from human tissue-culture cells. Proc. Natl Acad. Sci. U.S.A. 69, 452 10.1073/pnas.69.2.452 4501125 PMC426478

[60] Garcia-Gomez, S., Reyes, A., Martinez-Jimenez, M.I., Chocron, E.S., Mouron, S., Terrados, G. et al. (2013) PrimPol, an archaic primase/polymerase operating in human cells. Mol. Cell 52, 541–553 10.1016/j.molcel.2013.09.025 24207056 PMC3899013

[61] Bianchi, J., Rudd, S.G., Jozwiakowski, S.K., Bailey, L.J., Soura, V., Taylor, E. et al. (2013) Primpol bypasses UV photoproducts during eukaryotic chromosomal DNA replication. Mol. Cell 52, 566–573 10.1016/j.molcel.2013.10.035 24267451 PMC4228047

[62] Bailey, L.J. and Doherty, A.J. (2017) Mitochondrial DNA replication: a PrimPol perspective. Biochem. Soc. Trans. 45, 513–529 10.1042/BST20160162 28408491 PMC5390496

[63] Singh, B., Li, X.R., Owens, K.M., Vanniarajan, A., Liang, P. and Singh, K.K. (2015) Human REV3 DNA polymerase zeta localizes to mitochondria and protects the mitochondrial genome. PLoS One 10, e0140409 10.1371/journal.pone.0140409 26462070 PMC4604079

[64] Schreier, H.K., Wiehe, R.S., Ricchetti, M. and Wiesmüller, L. (2022) Polymerase ζ is involved in mitochondrial DNA maintenance processes in concert with APE1 activity. Genes 13, 879 10.3390/genes13050879 35627264 PMC9141751

[65] Wisnovsky, S., Jean, S.R. and Kelley, S.O. (2016) Mitochondrial DNA repair and replication proteins revealed by targeted chemical probes. Nat. Chem. Biol. 12, 567 10.1038/nchembio.2102 27239789

[66] Wisnovsky, S., Sack, T., Pagliarini, D.J., Laposa, R.R. and Kelley, S.O. (2018) DNA polymerase θ increases mutational rates in mitochondrial DNA. ACS Chem. Biol. 13, 900–908 10.1021/acschembio.8b00072 29509408 PMC5914477

[67] Sykora, P., Kanno, S., Akbari, M., Kulikowicz, T., Baptiste, B.A., Leandro, G.S. et al. (2017) DNA polymerase beta participates in mitochondrial DNA repair. Mol. Cell. Biol. 37, e00237-17 10.1128/MCB.00237-17 28559431 PMC5533889

[68] Prasad, R., Çaglayan, M., Dai, D.P., Nadalutti, C.A., Zhao, M.L., Gassman, N.R. et al. (2017) DNA polymerase β: a missing link of the base excision repair machinery in mammalian mitochondria. DNA Repair 60, 77–88 10.1016/j.dnarep.2017.10.011 29100041 PMC5919216

[69] Krasich, R. and Copeland, W.C. (2017) DNA polymerases in the mitochondria: a critical review of the evidence. Front. Biosci. 22, 692–709 10.2741/4510 PMC5485829 27814640

[70] Yakubovskaya, E., Chen, Z.X., Carrodeguas, J.A., Kisker, C. and Bogenhagen, D.F. (2006) Functional human mitochondrial DNA polymerase γ forms a heterotrimer. J. Biol. Chem. 281, 374–382 10.1074/jbc.M509730200 16263719

[71] Gray, H. and Wong, T.W. (1992) Purification and identification of subunit structure of the human mitochondrial-DNA polymerase. J. Biol. Chem. 267, 5835–5841 10.1016/S0021-9258(18)42629-4 1556099

[72] Carrodeguas, J.A., Kobayashi, R., Lim, S.E., Copeland, W.C. and Bogenhagen, D.F. (1999) The accessory subunit of mitochondrial DNA polymerase γ increases processivity of the catalytic subunit of human DNA polymerase γ and is related to class II aminoacyl-tRNA synthetases. Mol. Cell. Biol. 19, 4039–4046 10.1128/MCB.19.6.4039 10330144 PMC104363

[73] Carrodeguas, J.A., Pinz, K.G. and Bogenhagen, D.F. (2002) DNA binding properties of human pol γB. J. Biol. Chem. 277, 50008–50014 10.1074/jbc.M207030200 12379656

[74] Kaguni, L.S. (2004) DNA polymerase γ, the mitochondrial replicase. Annu. Rev. Biochem. 73, 293–320 10.1146/annurev.biochem.72.121801.161455 15189144

[75] Bedford, E., Tabor, S. and Richardson, C.C. (1997) The thioredoxin binding domain of bacteriophage T7 DNA polymerase confers processivity on *Escherichia coli* DNA polymerase I. Proc. Natl Acad. Sci. U.S.A. 94, 479–484 10.1073/pnas.94.2.479 9012809 PMC19538

[76] Carrodeguas, J.A., Theis, K., Bogenhagen, D.F. and Kisker, C. (2001) Crystal structure and deletion analysis show that the accessory subunit of mammalian DNA polymerase γ, PolγB, functions as a homodimer. Mol. Cell 7, 43–54 10.1016/S1097-2765(01)00153-8 11172710

[77] Fan, L., Kim, S., Farr, C.L., Schaefer, K.T., Randolph, K.M., Tainer, J.A. et al. (2006) A novel processive mechanism for DNA synthesis revealed by structure, modeling and mutagenesis of the accessory subunit of human mitochondrial DNA polymerase. J. Mol. Biol. 358, 1229–1243 10.1016/j.jmb.2006.02.073 16574152 PMC4703138

[78] Korhonen, J.A., Gaspari, M. and Falkenberg, M. (2003) TWINKLE has 5′→3′ DNA helicase activity and is specifically stimulated by mitochondrial single-stranded DNA-binding protein. J. Biol. Chem. 278, 48627–48632 10.1074/jbc.M306981200 12975372

[79] Spelbrink, J.N., Li, F.Y., Tiranti, V., Nikali, K., Yuan, Q.P., Tariq, M. e t al. (2001) Human mitochondrial DNA deletions associated with mutations in the gene encoding Twinkle, a phage T7 gene 4-like protein localized in mitochondria. Nat. Genet. 28, 223–231 10.1038/90058 11431692

[80] Shutt, T.E. and Gray, M.W. (2006) Bacteriophage origins of mitochondrial replication and transcription proteins. Trends Genet. 22, 90–95 10.1016/j.tig.2005.11.007 16364493

[81] Tiranti, V., Rocchi, M., Didonato, S. and Zeviani, M. (1993) Cloning of human and rat cDNAs encoding the mitochondrial single-stranded DNA-binding protein (SSB). Gene 126, 219–225 10.1016/0378-1119(93)90370-I 8482537

[82] Qian, Y.F. and Johnson, K.A. (2017) The human mitochondrial single-stranded DNA-binding protein displays distinct kinetics and thermodynamics of DNA binding and exchange. J. Biol. Chem. 292, 13068–13084 10.1074/jbc.M117.791392 28615444 PMC5546044

[83] Kaur, P., Longley, M.J., Pan, H., Wang, H. and Copeland, W.C. (2018) Single-molecule DREEM imaging reveals DNA wrapping around human mitochondrial single-stranded DNA binding protein. Nucleic Acids Res. 46, 11287–11302 10.1093/nar/gky875 30256971 PMC6265486

[84] Yang, C., Curth, U., Urbanke, C. and Kang, C.H. (1997) Crystal structure of human mitochondrial single stranded DNA binding protein at 2.4 angstrom resolution. Nat. Struct. Biol. 4, 153–157 10.1038/nsb0297-153 9033597

[85] Vandyck, E., Foury, F., Stillman, B. and Brill, S.J. (1992) A single-stranded-DNA binding-protein required for mitochondrial-DNA replication in Saccharomyces-cerevisiae is homologous to Escherichia-coli SSB. EMBO J. 11, 3421–3430 10.1002/j.1460-2075.1992.tb05421.x 1324172 PMC556877

[86] Miralles Fuste, J., Shi, Y., Wanrooij, S., Zhu, X., Jemt, E., Persson, O. et al. (2014) In vivo occupancy of mitochondrial single-stranded DNA binding protein supports the strand displacement mode of DNA replication. PLoS Genet. 10, e1004832 10.1371/journal.pgen.1004832 25474639 PMC4256270

[87] Farr, C.L., Wang, Y.X. and Kaguni, L.S. (1999) Functional interactions of mitochondrial DNA polymerase and single-stranded DNA-binding protein - template-primer DNA binding and initiation and elongation of DNA strand synthesis. J. Biol. Chem. 274, 14779–14785 10.1074/jbc.274.21.14779 10329675

[88] Menger, K.E., Chapman, J., Diaz-Maldonado, H., Khazeem, M.M., Deen, D., Erdinc, D. et al. (2022) Two type I topoisomerases maintain DNA topology in human mitochondria. Nucleic Acids Res. 50, 11154–11174 10.1093/nar/gkac857 36215039 PMC9638942

[89] Uhler, J.P. and Falkenberg, M. (2015) Primer removal during mammalian mitochondrial DNA replication. DNA Repair 34, 28–38 10.1016/j.dnarep.2015.07.003 26303841

[90] Kornblum, C., Nicholls, T.J., Haack, T.B., Scholer, S., Peeva, V., Danhauser, K. et al. (2013) Loss-of-function mutations in MGME1 impair mtDNA replication and cause multisystemic mitochondrial disease. Nat. Genet. 45, 214–219 10.1038/ng.2501 23313956 PMC3678843

[91] Szczesny, R.J., Hejnowicz, M.S., Steczkiewicz, K., Muszewska, A., Borowski, L.S., Ginalski, K. et al. (2013) Identification of a novel human mitochondrial endo-/exonuclease Ddk1/c20orf72 necessary for maintenance of proper 7S DNA levels. Nucleic Acids Res. 41, 3144–3161 10.1093/nar/gkt029 23358826 PMC3597694

[92] Macao, B., Uhler, J.P., Siibak, T., Zhu, X., Shi, Y., Sheng, W. et al. (2015) The exonuclease activity of DNA polymerase gamma is required for ligation during mitochondrial DNA replication. Nat. Commun. 6, 7303 10.1038/ncomms8303 26095671 PMC4557304

[93] Lakshmipathy, U. and Campbell, C. (1999) The human DNA ligase III gene encodes nuclear and mitochondrial proteins. Mol. Cell. Biol. 19, 3869–3876 10.1128/MCB.19.5.3869 10207110 PMC84244

[94] Simsek, D., Furda, A., Gao, Y., Artus, J., Brunet, E., Hadjantonakis, A.K. et al. (2011) Crucial role for DNA ligase III in mitochondria but not in Xrcc1-dependent repair. Nature 471, 245–248 10.1038/nature09794 21390132 PMC3261757

[95] Gao, Y.K., Katyal, S., Lee, Y.S., Zhao, J.F., Rehg, J.E., Russell, H.R. et al. (2011) DNA ligase III is critical for mtDNA integrity but not Xrcc1-mediated nuclear DNA repair. Nature 471, 240–244 10.1038/nature09773 21390131 PMC3079429

[96] Pommier, Y., Nussenzweig, A., Takeda, S. and Austin, C. (2022) Human topoisomerases and their roles in genome stability and organization. Nat. Rev. Mol. Cell Biol. 23, 407–427 10.1038/s41580-022-00452-3 35228717 PMC8883456

[97] Wang, Y., Lyu, Y.L. and Wang, J.C. (2002) Dual localization of human DNA topoisomerase IIIalpha to mitochondria and nucleus. Proc. Natl Acad. Sci. U.S.A. 99, 12114–12119 10.1073/pnas.192449499 12209014 PMC129407

[98] Yang, J., Bachrati, C.Z., Ou, J., Hickson, I.D. and Brown, G.W. (2010) Human topoisomerase IIIalpha is a single-stranded DNA decatenase that is stimulated by BLM and RMI1. J. Biol. Chem. 285, 21426–21436 10.1074/jbc.M110.123216 20445207 PMC2898442

[99] Li, W. and Wang, J.C. (1998) Mammalian DNA topoisomerase IIIα is essential in early embryogenesis. Proc. Natl Acad. Sci. U.S.A. 95, 1010–1013 10.1073/pnas.95.3.1010 9448276 PMC18654

[100] Nicholls, T.J., Nadalutti, C.A., Motori, E., Sommerville, E.W., Gorman, G.S., Basu, S. et al. (2018) Topoisomerase 3alpha is required for decatenation and segregation of human mtDNA. Mol. Cell 69, 9–23 e26 10.1016/j.molcel.2017.11.033 29290614 PMC5935120

[101] Zhang, H.L., Zhang, Y.W., Yasukawa, T., Dalla Rosa, I., Khiati, S. and Pommier, Y. (2014) Increased negative supercoiling of mtDNA in knockout mice and presence of topoisomerases IIα and IIβ in vertebrate mitochondria. Nucleic Acids Res. 42, 7259–7267 10.1093/nar/gku384 24803675 PMC4066791

[102] Hangas, A., Aasumets, K., Kekäläinen, N.J., Paloheinä, M., Pohjoismäki, J.L., Gerhold, J.M. et al. (2018) Ciprofloxacin impairs mitochondrial DNA replication initiation through inhibition of Topoisomerase 2. Nucleic Acids Res. 46, 9625–9636 10.1093/nar/gky793 30169847 PMC6182158

[103] Low, R.L., Orton, S. and Friedman, D.B. (2003) A truncated form of DNA topoisomerase IIβ associates with the mtDNA genome in mammalian mitochondria. Eur. J. Biochem. 270, 4173–4186 10.1046/j.1432-1033.2003.03814.x 14519130

[104] Menger, K.E., Rodriguez-Luis, A., Chapman, J. and Nicholls, T.J. (2021) Controlling the topology of mammalian mitochondrial DNA. Open Biol. 11, 210168 10.1098/rsob.210168 34547213 PMC8455175

[105] Gillum, A.M. and Clayton, D.A. (1979) Mechanism of mitochondrial DNA replication in mouse L-cells: RNA priming during the initiation of heavy-strand synthesis. J. Mol. Biol. 135, 353–368 10.1016/0022-2836(79)90441-8 537082

[106] Ojala, D., Crews, S., Montoya, J., Gelfand, R. and Attardi, G. (1981) A small polyadenylated RNA (7 S RNA), containing a putative ribosome attachment site, maps near the origin of human mitochondrial DNA replication. J. Mol. Biol. 150, 303–314 10.1016/0022-2836(81)90454-X 6172590

[107] Zhu, X., Xie, X., Das, H., Tan, B.G., Shi, Y., Al-Behadili, A. et al. (2022) Non-coding 7S RNA inhibits transcription via mitochondrial RNA polymerase dimerization. Cell 185, 2309–2323 e2324 10.1016/j.cell.2022.05.006 35662414

[108] Xu, B. and Clayton, D.A. (1995) A persistent RNA-DNA hybrid is formed during transcription at a phylogenetically conserved mitochondrial DNA sequence. Mol. Cell. Biol. 15, 580–589 10.1128/MCB.15.1.580 7528331 PMC232017

[109] Xu, B. and Clayton, D.A. (1996) RNA-DNA hybrid formation at the human mitochondrial heavy-strand origin ceases at replication start sites: an implication for RNA-DNA hybrids serving as primers. EMBO J. 15, 3135–3143 10.1002/j.1460-2075.1996.tb00676.x 8670814 PMC450256

[110] Wanrooij, P.H., Uhler, J.P., Shi, Y., Westerlund, F., Falkenberg, M. and Gustafsson, C.M. (2012) A hybrid G-quadruplex structure formed between RNA and DNA explains the extraordinary stability of the mitochondrial R-loop. Nucleic Acids Res. 40, 10334–10344 10.1093/nar/gks802 22965135 PMC3488243

[111] Chang, D.D. and Clayton, D.A. (1987) A novel endoribonuclease cleaves at a priming site of mouse mitochondrial DNA replication. EMBO J. 6, 409–417 10.1002/j.1460-2075.1987.tb04770.x 3582365 PMC553411

[112] Lee, D.Y. and Clayton, D.A. (1997) RNase mitochondrial RNA processing correctly cleaves a novel R loop at the mitochondrial DNA leading-strand origin of replication. Genes Dev. 11, 582–592 10.1101/gad.11.5.582 9119223

[113] Lee, D.Y. and Clayton, D.A. (1998) Initiation of mitochondrial DNA replication by transcription and R-loop processing. J. Biol. Chem. 273, 30614–30621 10.1074/jbc.273.46.30614 9804833

[114] Kiss, T. and Filipowicz, W. (1992) Evidence against a mitochondrial location of the 7-2/MRP RNA in mammalian cells. Cell 70, 11–16 10.1016/0092-8674(92)90528-K 1377982

[115] Kiss, T., Marshallsay, C. and Filipowicz, W. (1992) 7-2/MRP RNAs in plant and mammalian cells: association with higher order structures in the nucleolus. EMBO J. 11, 3737–3746 10.1002/j.1460-2075.1992.tb05459.x 1382978 PMC556834

[116] Gammage, P.A., Moraes, C.T. and Minczuk, M. (2018) Mitochondrial genome engineering: the revolution may not be CRISPR-Ized. Trends Genet. 34, 101–110 10.1016/j.tig.2017.11.001 29179920 PMC5783712

[117] Brown, T.A. and Clayton, D.A. (2002) Release of replication termination controls mitochondrial DNA copy number after depletion with 2′,3′-dideoxycytidine. Nucleic Acids Res. 30, 2004–2010 10.1093/nar/30.9.2004 11972339 PMC113833

[118] Bogenhagen, D. and Clayton, D.A. (1978) Mechanism of mitochondrial DNA replication in mouse L-cells: kinetics of synthesis and turnover of the initiation sequence. J. Mol. Biol. 119, 49–68 10.1016/0022-2836(78)90269-3 633368

[119] Agaronyan, K., Morozov, Y.I., Anikin, M. and Temiakov, D. (2015) Mitochondrial biology. Replication-transcription switch in human mitochondria. Science 347, 548–551 10.1126/science.aaa0986 25635099 PMC4677687

[120] Posse, V., Shahzad, S., Falkenberg, M., Hallberg, B.M. and Gustafsson, C.M. (2015) TEFM is a potent stimulator of mitochondrial transcription elongation in vitro. Nucleic Acids Res. 43, 2615–2624 10.1093/nar/gkv105 25690892 PMC4357710

[121] Pham, X.H., Farge, G., Shi, Y., Gaspari, M., Gustafsson, C.M. and Falkenberg, M. (2006) Conserved sequence box II directs transcription termination and primer formation in mitochondria. J. Biol. Chem. 281, 24647–24652 10.1074/jbc.M602429200 16790426

[122] Minczuk, M., He, J., Duch, A.M., Ettema, T.J., Chlebowski, A., Dzionek, K. et al. (2011) TEFM (c17orf42) is necessary for transcription of human mtDNA. Nucleic Acids Res. 39, 4284–4299 10.1093/nar/gkq1224 21278163 PMC3105396

[123] Jiang, S., Koolmeister, C., Misic, J., Siira, S., Kuhl, I., Silva Ramos, E. et al. (2019) TEFM regulates both transcription elongation and RNA processing in mitochondria. EMBO Rep. 20, e48101 10.15252/embr.201948101 31036713 PMC6549021

[124] Robberson, D.L., Kasamatsu, H. and Vinograd, J. (1972) Replication of mitochondrial DNA. Circular replicative intermediates in mouse L cells. Proc. Natl Acad. Sci. U.S.A. 69, 737–741 10.1073/pnas.69.3.737 4501588 PMC426547

[125] Fuste, J.M., Wanrooij, S., Jemt, E., Granycome, C.E., Cluett, T.J., Shi, Y. et al. (2010) Mitochondrial RNA polymerase is needed for activation of the origin of light-strand DNA replication. Mol. Cell 37, 67–78 10.1016/j.molcel.2009.12.021 20129056

[126] Sarfallah, A., Zamudio-Ochoa, A., Anikin, M. and Temiakov, D. (2021) Mechanism of transcription initiation and primer generation at the mitochondrial replication origin OriL. EMBO J. 40, e107988 10.15252/embj.2021107988 34423452 PMC8488568

[127] Wanrooij, S., Fusté, J.M., Farge, G., Shi, Y.H., Gustafsson, C.M. and Falkenberg, M. (2008) Human mitochondrial RNA polymerase primes lagging-strand DNA synthesis. Proc. Natl Acad. Sci. U.S.A. 105, 11122–11127 10.1073/pnas.0805399105 18685103 PMC2516254

[128] Wanrooij, S., Miralles Fuste, J., Stewart, J.B., Wanrooij, P.H., Samuelsson, T., Larsson, N.G. et al. (2012) In vivo mutagenesis reveals that OriL is essential for mitochondrial DNA replication. EMBO Rep. 13, 1130–1137 10.1038/embor.2012.161 23090476 PMC3513414

[129] Basu, S., Xie, X., Uhler, J.P., Hedberg-Oldfors, C., Milenkovic, D., Baris, O.R. et al. (2020) Accurate mapping of mitochondrial DNA deletions and duplications using deep sequencing. PLoS Genet. 16, e1009242 10.1371/journal.pgen.1009242 33315859 PMC7769605

[130] Liu, L.F. and Wang, J.C. (1987) Supercoiling of the DNA template during transcription. Proc. Natl Acad. Sci. U.S.A. 84, 7024–7027 10.1073/pnas.84.20.7024 2823250 PMC299221

[131] Peter, B.J., Ullsperger, C., Hiasa, H., Marians, K.J. and Cozzarelli, N.R. (1998) The structure of supercoiled intermediates in DNA replication. Cell 94, 819–827 10.1016/S0092-8674(00)81740-7 9753328

[132] Zhang, H., Barcelo, J.M., Lee, B., Kohlhagen, G., Zimonjic, D.B., Popescu, N.C. et al. (2001) Human mitochondrial topoisomerase I. Proc. Natl Acad. Sci. U.S.A. 98, 10608–10613 10.1073/pnas.191321998 11526219 PMC58513

[133] Sobek, S., Dalla Rosa, I., Pommier, Y., Bornholz, B., Kalfalah, F., Zhang, H. et al. (2013) Negative regulation of mitochondrial transcription by mitochondrial topoisomerase I. Nucleic Acids Res. 41, 9848–9857 10.1093/nar/gkt768 23982517 PMC3834834

[134] Dalla Rosa, I., Zhang, H., Khiati, S., Wu, X. and Pommier, Y. (2017) Transcription profiling suggests that mitochondrial topoisomerase IB acts as a topological barrier and regulator of mitochondrial DNA transcription. J. Biol. Chem. 292, 20162–20172 10.1074/jbc.M117.815241 29021209 PMC5724003

[135] Zhang, H. and Pommier, Y. (2008) Mitochondrial topoisomerase I sites in the regulatory D-loop region of mitochondrial DNA. Biochemistry 47, 11196–11203 10.1021/bi800774b 18826252 PMC2597090

[136] Dalla Rosa, I., Huang, S.Y., Agama, K., Khiati, S., Zhang, H. and Pommier, Y. (2014) Mapping topoisomerase sites in mitochondrial DNA with a poisonous mitochondrial topoisomerase I (Top1mt). J. Biol. Chem. 289, 18595–18602 10.1074/jbc.M114.555367 24798329 PMC4140277

[137] Bizard, A.H. and Hickson, I.D. (2020) The many lives of type IA topoisomerases. J. Biol. Chem. 295, 7138–7153 10.1074/jbc.REV120.008286 32277049 PMC7242696

[138] Saha, L.K. and Pommier, Y. (2024) TOP3A coupling with replication forks and repair of TOP3A cleavage complexes. Cell Cycle 23, 115–130 10.1080/15384101.2024.2314440 38341866 PMC11037291

[139] Hangas, A., Kekalainen, N.J., Potter, A., Michell, C., Aho, K.J., Rutanen, C. et al. (2022) Top3alpha is the replicative topoisomerase in mitochondrial DNA replication. Nucleic Acids Res. 50, 8733–8748 10.1093/nar/gkac660 35904803 PMC9410902

[140] Al-Behadili, A., Uhler, J.P., Berglund, A.K., Peter, B., Doimo, M., Reyes, A. et al. (2018) A two-nuclease pathway involving RNase H1 is required for primer removal at human mitochondrial OriL. Nucleic Acids Res. 46, 9471–9483 10.1093/nar/gky708 30102370 PMC6182146

[141] Uhler, J.P., Thorn, C., Nicholls, T.J., Matic, S., Milenkovic, D., Gustafsson, C.M. et al. (2016) MGME1 processes flaps into ligatable nicks in concert with DNA polymerase gamma during mtDNA replication. Nucleic Acids Res. 44, 5861–5871 10.1093/nar/gkw468 27220468 PMC4937333

[142] Radloff, R., Bauer, W. and Vinograd, J. (1967) A dye-buoyant-density method for the detection and isolation of closed circular duplex DNA: the closed circular DNA in HeLa cells. Proc. Natl Acad. Sci. U.S.A. 57, 1514–1521 10.1073/pnas.57.5.1514 5231757 PMC224502

[143] Takamatsu, C., Umeda, S., Ohsato, T., Ohno, T., Abe, Y., Fukuoh, A. et al. (2002) Regulation of mitochondrial D-loops by transcription factor A and single-stranded DNA-binding protein. EMBO Rep. 3, 451–456 10.1093/embo-reports/kvf099 11964388 PMC1084112

[144] Clayton, D.A. (1982) Replication of animal mitochondrial DNA. Cell 28, 693–705 10.1016/0092-8674(82)90049-6 6178513

[145] Berk, A.J. and Clayton, D.A. (1974) Mechanism of mitochondrial DNA replication in mouse L-cells: asynchronous replication of strands, segregation of circular daughter molecules, aspects of topology and turnover of an initiation sequence. J. Mol. Biol. 86, 801–824 10.1016/0022-2836(74)90355-6 4473554

[146] Holt, I.J., Lorimer, H.E. and Jacobs, H.T. (2000) Coupled leading- and lagging-strand synthesis of mammalian mitochondrial DNA. Cell 100, 515–524 10.1016/S0092-8674(00)80688-1 10721989

[147] Pohjoismaki, J.L., Holmes, J.B., Wood, S.R., Yang, M.Y., Yasukawa, T., Reyes, A. et al. (2010) Mammalian mitochondrial DNA replication intermediates are essentially duplex but contain extensive tracts of RNA/DNA hybrid. J. Mol. Biol. 397, 1144–1155 10.1016/j.jmb.2010.02.029 20184890 PMC2857715

[148] Pohjoismaki, J.L., Goffart, S. and Spelbrink, J.N. (2011) Replication stalling by catalytically impaired Twinkle induces mitochondrial DNA rearrangements in cultured cells. Mitochondrion 11, 630–634 10.1016/j.mito.2011.04.002 21540127

[149] Bowmaker, M., Yang, M.Y., Yasukawa, T., Reyes, A., Jacobs, H.T., Huberman, J.A. et al. (2003) Mammalian mitochondrial DNA replicates bidirectionally from an initiation zone. J. Biol. Chem. 278, 50961–50969 10.1074/jbc.M308028200 14506235

[150] Brown, T.A., Cecconi, C., Tkachuk, A.N., Bustamante, C. and Clayton, D.A. (2005) Replication of mitochondrial DNA occurs by strand displacement with alternative light-strand origins, not via a strand-coupled mechanism. Genes Dev. 19, 2466–2476 10.1101/gad.1352105 16230534 PMC1257401

[151] Yang, M.Y., Bowmaker, M., Reyes, A., Vergani, L., Angeli, P., Gringeri, E. et al. (2002) Biased incorporation of ribonucleotides on the mitochondrial L-strand accounts for apparent strand-asymmetric DNA replication. Cell 111, 495–505 10.1016/S0092-8674(02)01075-9 12437923

[152] Yasukawa, T., Reyes, A., Cluett, T.J., Yang, M.Y., Bowmaker, M., Jacobs, H.T. et al. (2006) Replication of vertebrate mitochondrial DNA entails transient ribonucleotide incorporation throughout the lagging strand. EMBO J. 25, 5358–5371 10.1038/sj.emboj.7601392 17066082 PMC1636616

[153] Reyes, A., Kazak, L., Wood, S.R., Yasukawa, T., Jacobs, H.T. and Holt, I.J. (2013) Mitochondrial DNA replication proceeds via a ‘bootlace’ mechanism involving the incorporation of processed transcripts. Nucleic Acids Res. 41, 5837–5850 10.1093/nar/gkt196 23595151 PMC3675460

[154] Cluett, T.J., Akman, G., Reyes, A., Kazak, L., Mitchell, A., Wood, S.R. et al. (2018) Transcript availability dictates the balance between strand-asynchronous and strand-coupled mitochondrial DNA replication. Nucleic Acids Res. 46, 10771–10781 10.1093/nar/gky852 30239839 PMC6237803

[155] Yasukawa, T., Yang, M.Y., Jacobs, H.T. and Holt, I.J. (2005) A bidirectional origin of replication maps to the major noncoding region of human mitochondrial DNA. Mol. Cell 18, 651–662 10.1016/j.molcel.2005.05.002 15949440

[156] Robberson, D.L., Clayton, D.A. and Morrow, J.F. (1974) Cleavage of replicating forms of mitochondrial DNA by EcoRI endonuclease. Proc. Natl Acad. Sci. U.S.A. 71, 4447–4451 10.1073/pnas.71.11.4447 4612520 PMC433903

[157] Holt, I.J. and Reyes, A. (2012) Human mitochondrial DNA replication. Cold Spring Harb. Perspect. Biol. 4, a012971 10.1101/cshperspect.a012971 23143808 PMC3504440

[158] Samuels, D.C., Schon, E.A. and Chinnery, P.F. (2004) Two direct repeats cause most human mtDNA deletions. Trends Genet. 20, 393–398 10.1016/j.tig.2004.07.003 15313545

[159] Chen, T., He, J., Huang, Y. and Zhao, W. (2011) The generation of mitochondrial DNA large-scale deletions in human cells. J. Hum. Genet. 56, 689–694 10.1038/jhg.2011.97 21866113

[160] Mita, S., Rizzuto, R., Moraes, C.T., Shanske, S., Arnaudo, E., Fabrizi, G.M. et al. (1990) Recombination via flanking direct repeats is a major cause of large-scale deletions of human mitochondrial DNA. Nucleic Acids Res. 18, 561–567 10.1093/nar/18.3.561 2308845 PMC333462

[161] Fontana, G.A. and Gahlon, H.L. (2020) Mechanisms of replication and repair in mitochondrial DNA deletion formation. Nucleic Acids Res. 48, 11244–11258 10.1093/nar/gkaa804 33021629 PMC7672454

[162] Guo, X., Popadin, K.Y., Markuzon, N., Orlov, Y.L., Kraytsberg, Y., Krishnan, K.J. et al. (2010) Repeats, longevity and the sources of mtDNA deletions: evidence from ‘deletional spectra’. Trends Genet. 26, 340–343 10.1016/j.tig.2010.05.006 20591530 PMC2915442

[163] Persson, O., Muthukumar, Y., Basu, S., Jenninger, L., Uhler, J.P., Berglund, A.K. et al. (2019) Copy-choice recombination during mitochondrial L-strand synthesis causes DNA deletions. Nat. Commun. 10, 759 10.1038/s41467-019-08673-5 30770810 PMC6377680

[164] Bua, E., Johnson, J., Herbst, A., Delong, B., McKenzie, D., Salamat, S. et al. (2006) Mitochondrial DNA-deletion mutations accumulate intracellularly to detrimental levels in aged human skeletal muscle fibers. Am. J. Hum. Genet. 79, 469–480 10.1086/507132 16909385 PMC1559550

[165] Reeve, A.K., Krishnan, K.J., Elson, J.L., Morris, C.M., Bender, A., Lightowlers, R.N. et al. (2008) Nature of mitochondrial DNA deletions in substantia nigra neurons. Am. J. Hum. Genet. 82, 228–235 10.1016/j.ajhg.2007.09.018 18179904 PMC2253975

[166] Degoul, F., Nelson, I., Amselem, S., Romero, N., Obermaier-Kusser, B., Ponsot, G. et al. (1991) Different mechanisms inferred from sequences of human mitochondrial DNA deletions in ocular myopathies. Nucleic Acids Res. 19, 493–496 10.1093/nar/19.3.493 2011523 PMC333638

[167] Ruiz-Pesini, E., Lott, M.T., Procaccio, V., Poole, J.C., Brandon, M.C., Mishmar, D. et al. (2007) An enhanced MITOMAP with a global mtDNA mutational phylogeny. Nucleic Acids Res. 35, D823–D828 10.1093/nar/gkl927 17178747 PMC1781213

[168] Damas, J., Carneiro, J., Amorim, A. and Pereira, F. (2014) Mitobreak: the mitochondrial DNA breakpoints database. Nucleic Acids Res. 42, D1261–D1268 10.1093/nar/gkt982 24170808 PMC3965124

[169] Schon, E.A., Rizzuto, R., Moraes, C.T., Nakase, H., Zeviani, M. and DiMauro, S. (1989) A direct repeat is a hotspot for large-scale deletion of human mitochondrial DNA. Science 244, 346–349 10.1126/science.2711184 2711184

[170] Shoffner, J.M., Lott, M.T., Voljavec, A.S., Soueidan, S.A., Costigan, D.A. and Wallace, D.C. (1989) Spontaneous Kearns-Sayre/chronic external ophthalmoplegia plus syndrome associated with a mitochondrial DNA deletion: a slip-replication model and metabolic therapy. Proc. Natl Acad. Sci. U.S.A. 86, 7952–7956 10.1073/pnas.86.20.7952 2554297 PMC298190

[171] Lujan, S.A., Longley, M.J., Humble, M.H., Lavender, C.A., Burkholder, A., Blakely, E.L. et al. (2020) Ultrasensitive deletion detection links mitochondrial DNA replication, disease, and aging. Genome Biol. 21, 248 10.1186/s13059-020-02138-5 32943091 PMC7500033

[172] Cortopassi, G.A. and Arnheim, N. (1990) Detection of a specific mitochondrial DNA deletion in tissues of older humans. Nucleic Acids Res. 18, 6927–6933 10.1093/nar/18.23.6927 2263455 PMC332752

[173] Corral-Debrinski, M., Horton, T., Lott, M.T., Shoffner, J.M., Beal, M.F. and Wallace, D.C. (1992) Mitochondrial DNA deletions in human brain: regional variability and increase with advanced age. Nat. Genet. 2, 324–329 10.1038/ng1292-324 1303288

[174] Soong, N.W., Hinton, D.R., Cortopassi, G. and Arnheim, N. (1992) Mosaicism for a specific somatic mitochondrial DNA mutation in adult human brain. Nat. Genet. 2, 318–323 10.1038/ng1292-318 1303287

[175] Yen, T.C., Su, J.H., King, K.L. and Wei, Y.H. (1991) Ageing-associated 5 kb deletion in human liver mitochondrial DNA. Biochem. Biophys. Res. Commun. 178, 124–131 10.1016/0006-291X(91)91788-E 2069552

[176] Zhang, C., Baumer, A., Maxwell, R.J., Linnane, A.W. and Nagley, P. (1992) Multiple mitochondrial DNA deletions in an elderly human individual. FEBS Lett. 297, 34–38 10.1016/0014-5793(92)80321-7 1551433

[177] Simonetti, S., Chen, X., DiMauro, S. and Schon, E.A. (1992) Accumulation of deletions in human mitochondrial DNA during normal aging: analysis by quantitative PCR. Biochim. Biophys. Acta 1180, 113–122 10.1016/0925-4439(92)90059-V 1463763

[178] Zeviani, M., Servidei, S., Gellera, C., Bertini, E., DiMauro, S. and DiDonato, S. (1989) An autosomal dominant disorder with multiple deletions of mitochondrial DNA starting at the D-loop region. Nature 339, 309–311 10.1038/339309a0 2725645

[179] Erdinc, D., Rodriguez-Luis, A., Fassad, M.R., Mackenzie, S., Watson, C.M., Valenzuela, S. et al. (2023) Pathological variants in TOP3A cause distinct disorders of mitochondrial and nuclear genome stability. EMBO Mol. Med. 15, e16775 10.15252/emmm.202216775 37013609 PMC10165364

[180] Krishnan, K.J., Reeve, A.K., Samuels, D.C., Chinnery, P.F., Blackwood, J.K., Taylor, R.W. et al. (2008) What causes mitochondrial DNA deletions in human cells? Nat. Genet. 40, 275–279 10.1038/ng.f.94 18305478

[181] Wanrooij, S., Luoma, P., van Goethem, G., van Broeckhoven, C., Suomalainen, A. and Spelbrink, J.N. (2004) Twinkle and POLG defects enhance age-dependent accumulation of mutations in the control region of mtDNA. Nucleic Acids Res. 32, 3053–3064 10.1093/nar/gkh634 15181170 PMC434440

[182] Bharti, S.K., Sommers, J.A., Zhou, J., Kaplan, D.L., Spelbrink, J.N., Mergny, J.L. et al. (2014) DNA sequences proximal to human mitochondrial DNA deletion breakpoints prevalent in human disease form G-quadruplexes, a class of DNA structures inefficiently unwound by the mitochondrial replicative Twinkle helicase. J. Biol. Chem. 289, 29975–29993 10.1074/jbc.M114.567073 25193669 PMC4208006

[183] Dong, D.W., Pereira, F., Barrett, S.P., Kolesar, J.E., Cao, K., Damas, J. et al. (2014) Association of G-quadruplex forming sequences with human mtDNA deletion breakpoints. BMC Genomics 15, 677 10.1186/1471-2164-15-677 25124333 PMC4153896

[184] Doimo, M., Chaudhari, N., Abrahamsson, S., L'Hote, V., Nguyen, T.V.H., Berner, A. et al. (2023) Enhanced mitochondrial G-quadruplex formation impedes replication fork progression leading to mtDNA loss in human cells. Nucleic Acids Res. 51, 7392–7408 10.1093/nar/gkad535 37351621 PMC10415151

[185] Phillips, A.F., Millet, A.R., Tigano, M., Dubois, S.M., Crimmins, H., Babin, L. et al. (2017) Single-molecule analysis of mtDNA replication uncovers the basis of the common deletion. Mol. Cell 65, 527–538 e526 10.1016/j.molcel.2016.12.014 28111015

[186] Albertini, A.M., Hofer, M., Calos, M.P. and Miller, J.H. (1982) On the formation of spontaneous deletions: the importance of short sequence homologies in the generation of large deletions. Cell 29, 319–328 10.1016/0092-8674(82)90148-9 6288254

[187] Moretton, A., Morel, F., Macao, B., Lachaume, P., Ishak, L., Lefebvre, M. et al. (2017) Selective mitochondrial DNA degradation following double-strand breaks. PLoS One 12, e0176795 10.1371/journal.pone.0176795 28453550 PMC5409072

[188] Peeva, V., Blei, D., Trombly, G., Corsi, S., Szukszto, M.J., Rebelo-Guiomar, P. et al. (2018) Linear mitochondrial DNA is rapidly degraded by components of the replication machinery. Nat. Commun. 9, 1727 10.1038/s41467-018-04131-w 29712893 PMC5928156

[189] Nissanka, N., Bacman, S.R., Plastini, M.J. and Moraes, C.T. (2018) The mitochondrial DNA polymerase gamma degrades linear DNA fragments precluding the formation of deletions. Nat. Commun. 9, 2491 10.1038/s41467-018-04895-1 29950568 PMC6021392

[190] Bayona-Bafaluy, M.P., Blits, B., Battersby, B.J., Shoubridge, E.A. and Moraes, C.T. (2005) Rapid directional shift of mitochondrial DNA heteroplasmy in animal tissues by a mitochondrially targeted restriction endonuclease. Proc. Natl Acad. Sci. U.S.A. 102, 14392–14397 10.1073/pnas.0502896102 16179392 PMC1242285

[191] Srivastava, S. and Moraes, C.T. (2005) Double-strand breaks of mouse muscle mtDNA promote large deletions similar to multiple mtDNA deletions in humans. Hum. Mol. Genet. 14, 893–902 10.1093/hmg/ddi082 15703189 PMC1242110

[192] Fukui, H. and Moraes, C.T. (2009) Mechanisms of formation and accumulation of mitochondrial DNA deletions in aging neurons. Hum. Mol. Genet. 18, 1028–1036 10.1093/hmg/ddn437 19095717 PMC2722231

[193] Bacman, S.R., Williams, S.L. and Moraes, C.T. (2009) Intra- and inter-molecular recombination of mitochondrial DNA after in vivo induction of multiple double-strand breaks. Nucleic Acids Res. 37, 4218–4226 10.1093/nar/gkp348 19435881 PMC2715231

[194] Tadi, S.K., Sebastian, R., Dahal, S., Babu, R.K., Choudhary, B. and Raghavan, S.C. (2016) Microhomology-mediated end joining is the principal mediator of double-strand break repair during mitochondrial DNA lesions. Mol. Biol. Cell 27, 223–235 10.1091/mbc.e15-05-0260 26609070 PMC4713127

[195] Nissanka, N., Minczuk, M. and Moraes, C.T. (2019) Mechanisms of mitochondrial DNA deletion formation. Trends Genet. 35, 235–244 10.1016/j.tig.2019.01.001 30691869

[196] Hagstrom, E., Freyer, C., Battersby, B.J., Stewart, J.B. and Larsson, N.G. (2014) No recombination of mtDNA after heteroplasmy for 50 generations in the mouse maternal germline. Nucleic Acids Res. 42, 1111–1116 10.1093/nar/gkt969 24163253 PMC3902947

[197] Constantinou, A., Davies, A.A. and West, S.C. (2001) Branch migration and Holliday junction resolution catalyzed by activities from mammalian cells. Cell 104, 259–268 10.1016/S0092-8674(01)00210-0 11207366

[198] Cejka, P. and Symington, L.S. (2021) DNA end resection: mechanism and control. Annu. Rev. Genet. 55, 285–307 10.1146/annurev-genet-071719-020312 34813349

[199] Trifunovic, A., Wredenberg, A., Falkenberg, M., Spelbrink, J.N., Rovio, A.T., Bruder, C.E. et al. (2004) Premature ageing in mice expressing defective mitochondrial DNA polymerase. Nature 429, 417–423 10.1038/nature02517 15164064

[200] Bailey, L.J., Cluett, T.J., Reyes, A., Prolla, T.A., Poulton, J., Leeuwenburgh, C. et al. (2009) Mice expressing an error-prone DNA polymerase in mitochondria display elevated replication pausing and chromosomal breakage at fragile sites of mitochondrial DNA. Nucleic Acids Res. 37, 2327–2335 10.1093/nar/gkp091 19244310 PMC2673436

[201] Nicholls, T.J., Zsurka, G., Peeva, V., Scholer, S., Szczesny, R.J., Cysewski, D. et al. (2014) Linear mtDNA fragments and unusual mtDNA rearrangements associated with pathological deficiency of MGME1 exonuclease. Hum. Mol. Genet. 23, 6147–6162 10.1093/hmg/ddu336 24986917 PMC4222359

[202] Matic, S., Jiang, M., Nicholls, T.J., Uhler, J.P., Dirksen-Schwanenland, C., Polosa, P.L. et al. (2018) Mice lacking the mitochondrial exonuclease MGME1 accumulate mtDNA deletions without developing progeria. Nat. Commun. 9, 1202 10.1038/s41467-018-03552-x 29572490 PMC5865154

[203] Poulton, J., Deadman, M.E. and Gardiner, R.M. (1989) Duplications of mitochondrial DNA in mitochondrial myopathy. Lancet 1, 236–240 10.1016/S0140-6736(89)91256-7 2563411

[204] Rotig, A., Bessis, J.L., Romero, N., Cormier, V., Saudubray, J.M., Narcy, P. et al. (1992) Maternally inherited duplication of the mitochondrial genome in a syndrome of proximal tubulopathy, diabetes mellitus, and cerebellar ataxia. Am. J. Hum. Genet. 50, 364–370 1531167 PMC1682469

[205] Dunbar, D.R., Moonie, P.A., Swingler, R.J., Davidson, D., Roberts, R. and Holt, I.J. (1993) Maternally transmitted partial direct tandem duplication of mitochondrial DNA associated with diabetes mellitus. Hum. Mol. Genet. 2, 1619–1624 10.1093/hmg/2.10.1619 8268914

[206] Williams, S.L., Huang, J., Edwards, Y.J., Ulloa, R.H., Dillon, L.M., Prolla, T.A. et al. (2010) The mtDNA mutation spectrum of the progeroid Polg mutator mouse includes abundant control region multimers. Cell Metab. 12, 675–682 10.1016/j.cmet.2010.11.012 21109200 PMC3175596

[207] Jeedigunta, S.P., Minenkova, A.V., Palozzi, J.M. and Hurd, T.R. (2021) Avoiding extinction: recent advances in understanding mechanisms of mitochondrial DNA purifying selection in the germline. Annu. Rev. Genomics Hum. Genet. 22, 55–80 10.1146/annurev-genom-121420-081805 34038145

[208] Stewart, J.B. and Chinnery, P.F. (2021) Extreme heterogeneity of human mitochondrial DNA from organelles to populations. Nat. Rev. Genet. 22, 106–118 10.1038/s41576-020-00284-x 32989265

[209] Glastad, R.C. and Johnston, I.G. (2023) Mitochondrial network structure controls cell-to-cell mtDNA variability generated by cell divisions. PLoS Comput. Biol. 19, e1010953 10.1371/journal.pcbi.1010953 36952562 PMC10072490

[210] Holt, I.J., Harding, A.E. and Morgan-Hughes, J.A. (1988) Deletions of muscle mitochondrial DNA in patients with mitochondrial myopathies. Nature 331, 717–719 10.1038/331717a0 2830540

[211] Holt, I.J., Harding, A.E., Cooper, J.M., Schapira, A.H., Toscano, A., Clark, J.B. et al. (1989) Mitochondrial myopathies: clinical and biochemical features of 30 patients with major deletions of muscle mitochondrial DNA. Ann. Neurol. 26, 699–708 10.1002/ana.410260603 2604380

[212] Solano, A., Gamez, J., Carod, F.J., Pineda, M., Playan, A., Lopez-Gallardo, E. et al. (2003) Characterisation of repeat and palindrome elements in patients harbouring single deletions of mitochondrial DNA. J. Med. Genet. 40, e86 10.1136/jmg.40.7.e86 12843335 PMC1735535

[213] Tang, Y., Schon, E.A., Wilichowski, E., Vazquez-Memije, M.E., Davidson, E. and King, M.P. (2000) Rearrangements of human mitochondrial DNA (mtDNA): new insights into the regulation of mtDNA copy number and gene expression. Mol. Biol. Cell 11, 1471–1485 10.1091/mbc.11.4.1471 10749943 PMC14860

[214] Kazama, T., Nakamura, T., Watanabe, M., Sugita, M. and Toriyama, K. (2008) Suppression mechanism of mitochondrial ORF79 accumulation by Rf1 protein in BT-type cytoplasmic male sterile rice. Plant J. 55, 619–628 10.1111/j.1365-313X.2008.03529.x 18435825

[215] Lawless, C., Greaves, L., Reeve, A.K., Turnbull, D.M. and Vincent, A.E. (2020) The rise and rise of mitochondrial DNA mutations. Open Biol. 10, 200061 10.1098/rsob.200061 32428418 PMC7276526

[216] Spinazzola, A., Perez-Rodriguez, D., Jezek, J. and Holt, I.J. (2024) Mitochondrial DNA competition: starving out the mutant genome. Trends Pharmacol. Sci. 45, 225–242 10.1016/j.tips.2024.01.011 38402076

[217] Howell, N. (1983) Origin, cellular expression, and cybrid transmission of mitochondrial CAP-R, PYR-IND, and OLI-R mutant phenotypes. Somatic Cell Genet. 9, 1–24 10.1007/BF01544045 6836447

[218] Frascarelli, C., Zanetti, N., Nasca, A., Izzo, R., Lamperti, C., Lamantea, E. et al. (2023) Nanopore long-read next-generation sequencing for detection of mitochondrial DNA large-scale deletions. Front Genet. 14, 1089956 10.3389/fgene.2023.1089956 37456669 PMC10344361

[219] Vandiver, A.R., Pielstick, B., Gilpatrick, T., Hoang, A.N., Vernon, H.J., Wanagat, J. et al. (2022) Long read mitochondrial genome sequencing using Cas9-guided adaptor ligation. Mitochondrion 65, 176–183 10.1016/j.mito.2022.06.003 35787470 PMC9399971

[220] Vandiver, A.R., Hoang, A.N., Herbst, A., Lee, C.C., Aiken, J.M., McKenzie, D. et al. (2023) Nanopore sequencing identifies a higher frequency and expanded spectrum of mitochondrial DNA deletion mutations in human aging. Aging Cell 22, e13842 10.1111/acel.13842 37132288 PMC10265159

[221] Muraki, K., Nishimura, S., Goto, Y., Nonaka, I., Sakura, N. and Ueda, K. (1997) The association between haematological manifestation and mtDNA deletions in Pearson syndrome. J. Inherit. Metab. Dis. 20, 697–703 10.1023/A:1005378527077 9323565

[222] Yanagihara, I., Inui, K., Yanagihara, K., Park, Y.D., Tanaka, J., Ozono, K. et al. (2001) Fluorescence in situ hybridization analysis of peripheral blood cells in Pearson marrow-pancreas syndrome. J. Pediatr. 139, 452–455 10.1067/mpd.2001.116296 11562629

[223] Taylor, R.W., Barron, M.J., Borthwick, G.M., Gospel, A., Chinnery, P.F., Samuels, D.C. et al. (2003) Mitochondrial DNA mutations in human colonic crypt stem cells. J. Clin. Invest. 112, 1351–1360 10.1172/JCI19435 14597761 PMC228466

[224] Fan, W., Waymire, K.G., Narula, N., Li, P., Rocher, C., Coskun, P.E. et al. (2008) A mouse model of mitochondrial disease reveals germline selection against severe mtDNA mutations. Science 319, 958–962 10.1126/science.1147786 18276892 PMC3049809

[225] Stewart, J.B., Freyer, C., Elson, J.L., Wredenberg, A., Cansu, Z., Trifunovic, A. et al. (2008) Strong purifying selection in transmission of mammalian mitochondrial DNA. PLoS Biol. 6, e10 10.1371/journal.pbio.0060010 18232733 PMC2214808

[226] Burr, S.P., Pezet, M. and Chinnery, P.F. (2018) Mitochondrial DNA heteroplasmy and purifying selection in the mammalian female germ line. Dev. Growth Differ. 60, 21–32 10.1111/dgd.12420 29363102 PMC11520955

[227] Chinnery, P.F., DiMauro, S., Shanske, S., Schon, E.A., Zeviani, M., Mariotti, C. et al. (2004) Risk of developing a mitochondrial DNA deletion disorder. Lancet 364, 592–596 10.1016/S0140-6736(04)16851-7 15313359

[228] Inoue, K., Nakada, K., Ogura, A., Isobe, K., Goto, Y., Nonaka, I. et al. (2000) Generation of mice with mitochondrial dysfunction by introducing mouse mtDNA carrying a deletion into zygotes. Nat. Genet. 26, 176–181 10.1038/82826 11017072

[229] Ferreira, C.R., Rahman, S., Keller, M., Zschocke, J. and Group, I.A. (2021) An international classification of inherited metabolic disorders (ICIMD). J. Inherit. Metab. Dis. 44, 164–177 10.1002/jimd.12348 33340416 PMC9021760

[230] Frazier, A.E., Thorburn, D.R. and Compton, A.G. (2019) Mitochondrial energy generation disorders: genes, mechanisms, and clues to pathology. J. Biol. Chem. 294, 5386–5395 10.1074/jbc.R117.809194 29233888 PMC6462508

[231] Pitceathly, R.D., Rahman, S. and Hanna, M.G. (2012) Single deletions in mitochondrial DNA–molecular mechanisms and disease phenotypes in clinical practice. Neuromuscul. Disord. 22, 577–586 10.1016/j.nmd.2012.03.009 22578526

[232] Ahmed, N., Ronchi, D. and Comi, G.P. (2015) Genes and pathways involved in adult onset disorders featuring muscle mitochondrial DNA instability. Int. J. Mol. Sci. 16, 18054–18076 10.3390/ijms160818054 26251896 PMC4581235

[233] Mancuso, M., Orsucci, D., Angelini, C., Bertini, E., Carelli, V., Comi, G.P. et al. (2015) Redefining phenotypes associated with mitochondrial DNA single deletion. J. Neurol. 262, 1301–1309 10.1007/s00415-015-7710-y 25808502

[234] Broomfield, A., Sweeney, M.G., Woodward, C.E., Fratter, C., Morris, A.M., Leonard, J.V. et al. (2015) Paediatric single mitochondrial DNA deletion disorders: an overlapping spectrum of disease. J. Inherit. Metab. Dis. 38, 445–457 10.1007/s10545-014-9778-4 25352051 PMC4432108

[235] Keshavan, N. and Rahman, S. (2018) Natural history of mitochondrial disorders: a systematic review. Essays Biochem. 62, 423–442 10.1042/EBC20170108 29980629

[236] Yamashita, S., Nishino, I., Nonaka, I. and Goto, Y.I. (2008) Genotype and phenotype analyses in 136 patients with single large-scale mitochondrial DNA deletions. J. Hum. Genet. 53, 598 10.1007/s10038-008-0289-8 18414780

[237] Conti, F., Di Martino, S., Drago, F., Bucolo, C., Micale, V., Montano, V. et al. (2023) Red flags in primary mitochondrial diseases: what should we recognize? Int. J. Mol. Sci. 24, 16746 10.3390/ijms242316746 38069070 PMC10706469

[238] Grady, J.P., Campbell, G., Ratnaike, T., Blakely, E.L., Falkous, G., Nesbitt, V. et al. (2014) Disease progression in patients with single, large-scale mitochondrial DNA deletions. Brain 137, 323–334 10.1093/brain/awt321 24277717 PMC3914470

[239] Manea, E.M., Leverger, G., Bellmann, F., Stanescu, P.A., Mircea, A., Lebre, A.S. et al. (2009) Pearson syndrome in the neonatal period: two case reports and review of the literature. J. Pediatr. Hematol. Oncol. 31, 947–951 10.1097/MPH.0b013e3181bbc4ef 19881395

[240] Yoshimi, A., Ishikawa, K., Niemeyer, C. and Grunert, S.C. (2022) Pearson syndrome: a multisystem mitochondrial disease with bone marrow failure. Orphanet J. Rare Dis. 17, 379 10.1186/s13023-022-02538-9 36253820 PMC9575259

[241] Farruggia, P., Di Cataldo, A., Pinto, R.M., Palmisani, E., Macaluso, A., Valvo, L.L. et al. (2016) Pearson syndrome: a retrospective cohort study from the Marrow Failure Study Group of A.I.E.O.P. (Associazione Italiana Emato-Oncologia Pediatrica). JIMD Rep. 26, 37–43 10.1007/8904_2015_470 26238250 PMC4864774

[242] Gorman, G.S., Schaefer, A.M., Ng, Y., Gomez, N., Blakely, E.L., Alston, C.L. et al. (2015) Prevalence of nuclear and mitochondrial DNA mutations related to adult mitochondrial disease. Ann. Neurol. 77, 753–759 10.1002/ana.24362 25652200 PMC4737121

[243] Orsucci, D., Angelini, C., Bertini, E., Carelli, V., Comi, G.P., Federico, A. et al. (2017) Revisiting mitochondrial ocular myopathies: a study from the Italian Network. J. Neurol. 264, 1777–1784 10.1007/s00415-017-8567-z 28695364

[244] Rahman, S. and Copeland, W.C. (2019) POLG-related disorders and their neurological manifestations. Nat. Rev. Neurol. 15, 40–52 10.1038/s41582-018-0101-0 30451971 PMC8796686

[245] Whittaker, R.G., Devine, H.E., Gorman, G.S., Schaefer, A.M., Horvath, R., Ng, Y. et al. (2015) Epilepsy in adults with mitochondrial disease: a cohort study. Ann. Neurol. 78, 949–957 10.1002/ana.24525 26381753 PMC4737309

[246] Hikmat, O., Tzoulis, C., Chong, W.K., Chentouf, L., Klingenberg, C., Fratter, C. et al. (2017) The clinical spectrum and natural history of early-onset diseases due to DNA polymerase gamma mutations. Genet. Med. 19, 1217–1225 10.1038/gim.2017.35 28471437

[247] Harding, B.N. (1990) Progressive neuronal degeneration of childhood with liver disease (Alpers-Huttenlocher syndrome): a personal review. J. Child Neurol. 5, 273–287 10.1177/088307389000500402 2246481

[248] Fellman, V. and Kotarsky, H. (2011) Mitochondrial hepatopathies in the newborn period. Semin Fetal Neonatal Med. 16, 222–228 10.1016/j.siny.2011.05.002 21680270

[249] Vara, R., Pinon, M., Fratter, C., Hegarty, R. and Hadzic, N. (2023) Hepatic presentations of mitochondrial DNA depletion syndrome in children: a single tertiary liver centre experience. J. Inherit. Metab. Dis. 46, 634–648 10.1002/jimd.12633 37204315

[250] Bugiardini, E., Poole, O.V., Manole, A., Pittman, A.M., Horga, A., Hargreaves, I. et al. (2017) Clinicopathologic and molecular spectrum of RNASEH1-related mitochondrial disease. Neurol. Genet. 3, e149 10.1212/NXG.0000000000000149 28508084 PMC5413961

[251] Woodbridge, P., Liang, C., Davis, R.L., Vandebona, H. and Sue, C.M. (2013) POLG mutations in Australian patients with mitochondrial disease. Intern. Med. J. 43, 150–156 10.1111/j.1445-5994.2012.02847.x 22647225

[252] Van Goethem, G., Dermaut, B., Lofgren, A., Martin, J.J. and Van Broeckhoven, C. (2001) Mutation of POLG is associated with progressive external ophthalmoplegia characterized by mtDNA deletions. Nat. Genet. 28, 211–212 10.1038/90034 11431686

[253] Longley, M.J., Clark, S., Yu Wai Man, C., Hudson, G., Durham, S.E., Taylor, R.W. et al. (2006) Mutant POLG2 disrupts DNA polymerase gamma subunits and causes progressive external ophthalmoplegia. Am. J. Hum. Genet. 78, 1026–1034 10.1086/504303 16685652 PMC1474082

[254] Walter, M.C., Czermin, B., Muller-Ziermann, S., Bulst, S., Stewart, J.D., Hudson, G. et al. (2010) Late-onset ptosis and myopathy in a patient with a heterozygous insertion in POLG2. J. Neurol. 257, 1517–1523 10.1007/s00415-010-5565-9 20405137

[255] Borsche, M., Dulovic-Mahlow, M., Baumann, H., Tunc, S., Luth, T., Schaake, S. et al. (2024) POLG2-linked mitochondrial disease: functional insights from new mutation carriers and review of the literature. Cerebellum 23, 479–488 10.1007/s12311-023-01557-x 37085601 PMC10951043

[256] Dosekova, P., Dubiel, A., Karlowicz, A., Zietkiewicz, S., Rydzanicz, M., Habalova, V. et al. (2020) Whole exome sequencing identifies a homozygous POLG2 missense variant in an adult patient presenting with optic atrophy, movement disorders, premature ovarian failure and mitochondrial DNA depletion. Eur. J. Med. Genet. 63, 103821 10.1016/j.ejmg.2019.103821 31778857

[257] Carreno-Gago, L., Blazquez-Bermejo, C., Diaz-Manera, J., Camara, Y., Gallardo, E., Marti, R. et al. (2019) Identification and characterization of new RNASEH1 mutations associated with PEO syndrome and multiple mitochondrial DNA deletions. Front. Genet. 10, 576 10.3389/fgene.2019.00576 31258551 PMC6588129

[258] Manini, A., Caporali, L., Meneri, M., Zanotti, S., Piga, D., Arena, I.G. et al. (2022) Case report: rare homozygous RNASEH1 mutations associated with adult-onset mitochondrial encephalomyopathy and multiple mitochondrial DNA deletions. Front. Genet. 13, 906667 10.3389/fgene.2022.906667 35711919 PMC9194440

[259] Primiano, G., Torraco, A., Verrigni, D., Sabino, A., Bertini, E., Carrozzo, R. et al. (2022) Novel TOP3A variant associated with mitochondrial disease: expanding the clinical spectrum of topoisomerase III alpha-related diseases. Neurol. Genet. 8, e200007 10.1212/NXG.0000000000200007 35812164 PMC9258978

[260] Llaurado, A., Rovira-Moreno, E., Codina-Sola, M., Martinez-Saez, E., Salvado, M., Sanchez-Tejerina, D. et al. (2023) Chronic progressive external ophthalmoplegia plus syndrome due to homozygous missense variant in TOP3A gene. Clin. Genet. 103, 492–494 10.1111/cge.14287 36544354

[261] Martin, C.A., Sarlos, K., Logan, C.V., Thakur, R.S., Parry, D.A., Bizard, A.H. et al. (2018) Mutations in TOP3A cause a Bloom syndrome-like disorder. Am. J. Hum. Genet. 103, 221–231 10.1016/j.ajhg.2018.07.001 30057030 PMC6080766

[262] Jiang, W., Jia, N., Guo, C., Wen, J., Wu, L., Ogi, T. et al. (2021) Predominant cellular mitochondrial dysfunction in the TOP3A gene-caused Bloom syndrome-like disorder. Biochim. Biophys. Acta Mol. Basis Dis. 1867, 166106 10.1016/j.bbadis.2021.166106 33631320

[263] da Silva Rocha, E.B., de Lima Rodrigues, K., Montouro, L.A.M., Coelho, E.N., Kouyoumdjian, J.A., Kok, F. et al. (2023) A case of mitochondrial DNA depletion syndrome type 11 - expanding the genotype and phenotype. Neuromuscul. Disord. 33, 692–696 10.1016/j.nmd.2023.06.004 37429773

[264] Ronchi, D., Di Fonzo, A., Lin, W., Bordoni, A., Liu, C., Fassone, E. et al. (2013) Mutations in DNA2 link progressive myopathy to mitochondrial DNA instability. Am. J. Hum. Genet. 92, 293–300 10.1016/j.ajhg.2012.12.014 23352259 PMC3567272

[265] Gonzalez-Del Angel, A., Bisciglia, M., Vargas-Canas, S., Fernandez-Valverde, F., Kazakova, E., Escobar, R.E. et al. (2019) Novel phenotypes and cardiac involvement associated with DNA2 genetic variants. Front. Neurol. 10, 1049 10.3389/fneur.2019.01049 31636600 PMC6787284

[266] Phowthongkum, P. and Sun, A. (2017) Novel truncating variant in DNA2-related congenital onset myopathy and ptosis suggests genotype-phenotype correlation. Neuromuscul. Disord. 27, 616–618 10.1016/j.nmd.2017.03.013 28554558

[267] Stiles, A.R., Simon, M.T., Stover, A., Eftekharian, S., Khanlou, N., Wang, H.L. et al. (2016) Mutations in TFAM, encoding mitochondrial transcription factor A, cause neonatal liver failure associated with mtDNA depletion. Mol. Genet. Metab. 119, 91–99 10.1016/j.ymgme.2016.07.001 27448789

[268] Bourdon, A., Minai, L., Serre, V., Jais, J.P., Sarzi, E., Aubert, S. et al. (2007) Mutation of RRM2B, encoding p53-controlled ribonucleotide reductase (p53R2), causes severe mitochondrial DNA depletion. Nat. Genet. 39, 776–780 10.1038/ng2040 17486094

[269] Finsterer, J. and Zarrouk-Mahjoub, S. (2018) Phenotypic and genotypic heterogeneity of RRM2B variants. Neuropediatrics 49, 231–237 10.1055/s-0037-1609039 29241262

[270] Nishino, I., Spinazzola, A., Papadimitriou, A., Hammans, S., Steiner, I., Hahn, C.D. et al. (2000) Mitochondrial neurogastrointestinal encephalomyopathy: an autosomal recessive disorder due to thymidine phosphorylase mutations. Ann. Neurol. 47, 792–800 10.1002/1531-8249(200006)47:6<792::AID-ANA12>3.0.CO;2-Y 10852545

[271] Nishino, I., Spinazzola, A. and Hirano, M. (2001) MNGIE: from nuclear DNA to mitochondrial DNA. Neuromuscul. Disord. 11, 7–10 10.1016/S0960-8966(00)00159-0 11166160

[272] Nishino, I., Spinazzola, A. and Hirano, M. (1999) Thymidine phosphorylase gene mutations in MNGIE, a human mitochondrial disorder. Science 283, 689–692 10.1126/science.283.5402.689 9924029

[273] Saada, A., Shaag, A., Mandel, H., Nevo, Y., Eriksson, S. and Elpeleg, O. (2001) Mutant mitochondrial thymidine kinase in mitochondrial DNA depletion myopathy. Nat. Genet. 29, 342–344 10.1038/ng751 11687801

[274] Garone, C., Taylor, R.W., Nascimento, A., Poulton, J., Fratter, C., Dominguez-Gonzalez, C. et al. (2018) Retrospective natural history of thymidine kinase 2 deficiency. J. Med. Genet. 55, 515–521 10.1136/jmedgenet-2017-105012 29602790 PMC6073909

[275] Berardo, A., Dominguez-Gonzalez, C., Engelstad, K. and Hirano, M. (2022) Advances in thymidine kinase 2 deficiency: clinical aspects, translational progress, and emerging therapies. J. Neuromuscul. Dis. 9, 225–235 10.3233/JND-210786 35094997 PMC9028656

[276] Al-Hussaini, A., Faqeih, E., El-Hattab, A.W., Alfadhel, M., Asery, A., Alsaleem, B. et al. (2014) Clinical and molecular characteristics of mitochondrial DNA depletion syndrome associated with neonatal cholestasis and liver failure. J. Pediatr. 164, 553–559 e551–552 10.1016/j.jpeds.2013.10.082 24321534

[277] El-Hattab, A.W., Wang, J., Dai, H., Almannai, M., Staufner, C., Alfadhel, M. et al. (2018) MPV17-related mitochondrial DNA maintenance defect: new cases and review of clinical, biochemical, and molecular aspects. Hum. Mutat. 39, 461–470 10.1002/humu.23387 29282788

[278] Mandel, H., Szargel, R., Labay, V., Elpeleg, O., Saada, A., Shalata, A. et al. (2001) The deoxyguanosine kinase gene is mutated in individuals with depleted hepatocerebral mitochondrial DNA. Nat. Genet. 29, 337–341 10.1038/ng746 11687800

[279] Ronchi, D., Garone, C., Bordoni, A., Gutierrez Rios, P., Calvo, S.E., Ripolone, M. et al. (2012) Next-generation sequencing reveals DGUOK mutations in adult patients with mitochondrial DNA multiple deletions. Brain 135, 3404–3415 10.1093/brain/aws258 23043144 PMC3501975

[280] Buchaklian, A.H., Helbling, D., Ware, S.M. and Dimmock, D.P. (2012) Recessive deoxyguanosine kinase deficiency causes juvenile onset mitochondrial myopathy. Mol. Genet. Metab. 107, 92–94 10.1016/j.ymgme.2012.04.019 22622127

[281] Elpeleg, O., Miller, C., Hershkovitz, E., Bitner-Glindzicz, M., Bondi-Rubinstein, G., Rahman, S. et al. (2005) Deficiency of the ADP-forming succinyl-CoA synthase activity is associated with encephalomyopathy and mitochondrial DNA depletion. Am. J. Hum. Genet. 76, 1081–1086 10.1086/430843 15877282 PMC1196446

[282] Carrozzo, R., Verrigni, D., Rasmussen, M., de Coo, R., Amartino, H., Bianchi, M. et al. (2016) Succinate-CoA ligase deficiency due to mutations in SUCLA2 and SUCLG1: phenotype and genotype correlations in 71 patients. J. Inherit. Metab. Dis. 39, 243–252 10.1007/s10545-015-9894-9 26475597

[283] Ostergaard, E., Christensen, E., Kristensen, E., Mogensen, B., Duno, M., Shoubridge, E.A. et al. (2007) Deficiency of the alpha subunit of succinate-CoA ligase causes fatal infantile lactic acidosis with mtDNA depletion. Am. J. Hum. Genet. 81, 383–387 10.1086/519222 17668387 PMC1950792

[284] Besse, A., Wu, P., Bruni, F., Donti, T., Graham, B.H., Craigen, W.J. et al. (2015) The GABA transaminase, ABAT, is essential for mitochondrial nucleoside metabolism. Cell Metab. 21, 417–427 10.1016/j.cmet.2015.02.008 25738457 PMC4757431

[285] Jaeken, J., Casaer, P., de Cock, P., Corbeel, L., Eeckels, R., Eggermont, E. et al. (1984) Gamma-aminobutyric acid-transaminase deficiency: a newly recognized inborn error of neurotransmitter metabolism. Neuropediatrics 15, 165–169 10.1055/s-2008-1052362 6148708

[286] Koenig, M.K., Hodgeman, R., Riviello, J.J., Chung, W., Bain, J., Chiriboga, C.A. et al. (2017) Phenotype of GABA-transaminase deficiency. Neurology 88, 1919–1924 10.1212/WNL.0000000000003936 28411234 PMC5444310

[287] Finsterer, J. and Zarrouk-Mahjoub, S. (2018) Phenotypic spectrum of SLC25A4 mutations. Biomed. Rep. 9, 119–122 10.3892/br.2018.1115 30013777 PMC6036827

[288] Kaukonen, J., Juselius, J.K., Tiranti, V., Kyttala, A., Zeviani, M., Comi, G.P. et al. (2000) Role of adenine nucleotide translocator 1 in mtDNA maintenance. Science 289, 782–785 10.1126/science.289.5480.782 10926541

[289] Komaki, H., Fukazawa, T., Houzen, H., Yoshida, K., Nonaka, I. and Goto, Y. (2002) A novel D104G mutation in the adenine nucleotide translocator 1 gene in autosomal dominant progressive external ophthalmoplegia patients with mitochondrial DNA with multiple deletions. Ann. Neurol. 51, 645–648 10.1002/ana.10172 12112115

[290] Sengers, R.C., Trijbels, J.M., Willems, J.L., Daniels, O. and Stadhouders, A.M. (1975) Congenital cataract and mitochondrial myopathy of skeletal and heart muscle associated with lactic acidosis after exercise. J. Pediatr. 86, 873–880 10.1016/S0022-3476(75)80217-4 1168700

[291] Mayr, J.A., Haack, T.B., Graf, E., Zimmermann, F.A., Wieland, T., Haberberger, B. et al. (2012) Lack of the mitochondrial protein acylglycerol kinase causes Sengers syndrome. Am. J. Hum. Genet. 90, 314–320 10.1016/j.ajhg.2011.12.005 22284826 PMC3276657

[292] Wu, C.W., Caha, M., Smoot, L., Harris, D.J., Roberts, A.E., Sacharow, S. et al. (2023) Sengers syndrome and AGK-related disorders - minireview of phenotypic variability and clinical outcomes in molecularly confirmed cases. Mol. Genet. Metab. 139, 107626 10.1016/j.ymgme.2023.107626 37354892

[293] Amati-Bonneau, P., Valentino, M.L., Reynier, P., Gallardo, M.E., Bornstein, B., Boissiere, A. et al. (2008) OPA1 mutations induce mitochondrial DNA instability and optic atrophy ‘plus’ phenotypes. Brain 131, 338–351 10.1093/brain/awm298 18158317

[294] Yu-Wai-Man, P., Griffiths, P.G., Gorman, G.S., Lourenco, C.M., Wright, A.F., Auer-Grumbach, M. et al. (2010) Multi-system neurological disease is common in patients with OPA1 mutations. Brain 133, 771–786 10.1093/brain/awq007 20157015 PMC2842512

[295] Nasca, A., Rizza, T., Doimo, M., Legati, A., Ciolfi, A., Diodato, D. et al. (2017) Not only dominant, not only optic atrophy: expanding the clinical spectrum associated with OPA1 mutations. Orphanet. J. Rare Dis. 12, 89 10.1186/s13023-017-0641-1 28494813 PMC5427524

[296] Chen, H., Vermulst, M., Wang, Y.E., Chomyn, A., Prolla, T.A., McCaffery, J.M. et al. (2010) Mitochondrial fusion is required for mtDNA stability in skeletal muscle and tolerance of mtDNA mutations. Cell 141, 280–289 10.1016/j.cell.2010.02.026 20403324 PMC2876819

[297] Rouzier, C., Bannwarth, S., Chaussenot, A., Chevrollier, A., Verschueren, A., Bonello-Palot, N. et al. (2012) The MFN2 gene is responsible for mitochondrial DNA instability and optic atrophy ‘plus’ phenotype. Brain 135, 23–34 10.1093/brain/awr323 22189565

[298] Ma, Y., Sun, A., Zhang, Y., Fan, D. and Liu, X. (2021) The genotype and phenotype features in a large Chinese MFN2 mutation cohort. Front. Neurol. 12, 757518 10.3389/fneur.2021.757518 34721278 PMC8548668

[299] Pipis, M., Feely, S.M.E., Polke, J.M., Skorupinska, M., Perez, L., Shy, R.R. et al. (2020) Natural history of Charcot-Marie-Tooth disease type 2A: a large international multicentre study. Brain 143, 3589–3602 10.1093/brain/awaa323 33415332 PMC7805791

[300] Chung, K.W., Kim, S.B., Park, K.D., Choi, K.G., Lee, J.H., Eun, H.W. et al. (2006) Early onset severe and late-onset mild Charcot-Marie-Tooth disease with mitofusin 2 (MFN2) mutations. Brain 129, 2103–2118 10.1093/brain/awl174 16835246

[301] Bombelli, F., Stojkovic, T., Dubourg, O., Echaniz-Laguna, A., Tardieu, S., Larcher, K. et al. (2014) Charcot-Marie-Tooth disease type 2A: from typical to rare phenotypic and genotypic features. JAMA Neurol. 71, 1036–1042 10.1001/jamaneurol.2014.629 24957169

[302] Bonnen, P.E., Yarham, J.W., Besse, A., Wu, P., Faqeih, E.A., Al-Asmari, A.M. et al. (2013) Mutations in FBXL4 cause mitochondrial encephalopathy and a disorder of mitochondrial DNA maintenance. Am. J. Hum. Genet. 93, 773 10.1016/j.ajhg.2013.09.003 PMC3769921 23993193

[303] Gai, X., Ghezzi, D., Johnson, M.A., Biagosch, C.A., Shamseldin, H.E., Haack, T.B. et al. (2013) Mutations in FBXL4, encoding a mitochondrial protein, cause early-onset mitochondrial encephalomyopathy. Am. J. Hum. Genet. 93, 482–495 10.1016/j.ajhg.2013.07.016 23993194 PMC3769923

[304] El-Hattab, A.W., Dai, H., Almannai, M., Wang, J., Faqeih, E.A., Al Asmari, A. et al. (2017) Molecular and clinical spectra of FBXL4 deficiency. Hum. Mutat. 38, 1649–1659 10.1002/humu.23341 28940506

[305] Larsson, N.G., Holme, E., Kristiansson, B., Oldfors, A. and Tulinius, M. (1990) Progressive increase of the mutated mitochondrial DNA fraction in Kearns-Sayre syndrome. Pediatr. Res. 28, 131–136 10.1203/00006450-199008000-00011 2395603

[306] Vincent, A.E., Rosa, H.S., Pabis, K., Lawless, C., Chen, C., Grünewald, A. et al. (2018) Subcellular origin of mitochondrial DNA deletions in human skeletal muscle. Ann. Neurol. 84, 289–301 10.1002/ana.25288 30014514 PMC6141001

[307] El-Hattab, A.W. and Scaglia, F. (2013) Mitochondrial DNA depletion syndromes: review and updates of genetic basis, manifestations, and therapeutic options. Neurotherapeutics 10, 186–198 10.1007/s13311-013-0177-6 23385875 PMC3625391

[308] Campbell, G., Krishnan, K.J., Deschauer, M., Taylor, R.W. and Turnbull, D.M. (2014) Dissecting the mechanisms underlying the accumulation of mitochondrial DNA deletions in human skeletal muscle. Hum. Mol. Genet. 23, 4612–4620 10.1093/hmg/ddu176 24740879 PMC4119413

[309] Lax, N.Z., Gorman, G.S. and Turnbull, D.M. (2017) Review: central nervous system involvement in mitochondrial disease. Neuropathol. Appl. Neurobiol. 43, 102–118 10.1111/nan.12333 27287935 PMC5363248

[310] Ticci, C., Orsucci, D., Ardissone, A., Bello, L., Bertini, E., Bonato, I. et al. (2021) Movement disorders in children with a mitochondrial disease: a cross-sectional survey from the Nationwide Italian Collaborative Network of Mitochondrial Diseases. J. Clin. Med. 10, 2063 10.3390/jcm10102063 34065803 PMC8151313

[311] Tranchant, C. and Anheim, M. (2016) Movement disorders in mitochondrial diseases. Rev. Neurol. 172, 524–529 10.1016/j.neurol.2016.07.003 27476418

[312] Zeviani, M. and Carelli, V. (2021) Mitochondrial retinopathies. Int. J. Mol. Sci. 23, 210 10.3390/ijms23010210 35008635 PMC8745158

[313] Finsterer, J. and Frank, M. (2017) Gastrointestinal manifestations of mitochondrial disorders: a systematic review. Therap. Adv. Gastroenterol. 10, 142–154 10.1177/1756283X16666806 PMC5330602 28286566

[314] Al-Gadi, I.S., Haas, R.H., Falk, M.J., Goldstein, A. and McCormack, S.E. (2018) Endocrine disorders in primary mitochondrial disease. J. Endocr. Soc. 2, 361–373 10.1210/js.2017-00434 29594260 PMC5865537

[315] Schaefer, A.M., Walker, M., Turnbull, D.M. and Taylor, R.W. (2013) Endocrine disorders in mitochondrial disease. Mol. Cell. Endocrinol. 379, 2–11 10.1016/j.mce.2013.06.004 23769710 PMC3820028

[316] Imasawa, T., Hirano, D., Nozu, K., Kitamura, H., Hattori, M., Sugiyama, H. et al. (2022) Clinicopathologic features of mitochondrial nephropathy. Kidney Int. Rep. 7, 580–590 10.1016/j.ekir.2021.12.028 35257070 PMC8897298

[317] Govers, L.P., Toka, H.R., Hariri, A., Walsh, S.B. and Bockenhauer, D. (2021) Mitochondrial DNA mutations in renal disease: an overview. Pediatr. Nephrol. 36, 9–17 10.1007/s00467-019-04404-6 31925537 PMC7701126

[318] Ng, Y.S., Lax, N.Z., Blain, A.P., Erskine, D., Baker, M.R., Polvikoski, T. et al. (2022) Forecasting stroke-like episodes and outcomes in mitochondrial disease. Brain 145, 542–554 10.1093/brain/awab353 34927673 PMC9014738

[319] Rotig, A., Cormier, V., Blanche, S., Bonnefont, J.P., Ledeist, F., Romero, N. et al. (1990) Pearson's marrow-pancreas syndrome. A multisystem mitochondrial disorder in infancy. J. Clin. Invest. 86, 1601–1608 10.1172/JCI114881 2243133 PMC296909

[320] Moraes, C.T., DiMauro, S., Zeviani, M., Lombes, A., Shanske, S., Miranda, A.F. et al. (1989) Mitochondrial DNA deletions in progressive external ophthalmoplegia and Kearns-Sayre syndrome. N. Engl. J. Med. 320, 1293–1299 10.1056/NEJM198905183202001 2541333

[321] Moraes, C.T., Sciacco, M., Ricci, E., Tengan, C.H., Hao, H., Bonilla, E. et al. (1995) Phenotype-genotype correlations in skeletal muscle of patients with mtDNA deletions. Muscle Nerve Suppl. 3, S150–S153 10.1002/mus.880181429 7603517

[322] Lopez-Gallardo, E., Lopez-Perez, M.J., Montoya, J. and Ruiz-Pesini, E. (2009) CPEO and KSS differ in the percentage and location of the mtDNA deletion. Mitochondrion 9, 314–317 10.1016/j.mito.2009.04.005 19410662

[323] Rocha, M.C., Rosa, H.S., Grady, J.P., Blakely, E.L., He, L., Romain, N. et al. (2018) Pathological mechanisms underlying single large-scale mitochondrial DNA deletions. Ann. Neurol. 83, 115–130 10.1002/ana.25127 29283441 PMC5893934

[324] Rusecka, J., Kaliszewska, M., Bartnik, E. and Tonska, K. (2018) Nuclear genes involved in mitochondrial diseases caused by instability of mitochondrial DNA. J. Appl. Genet. 59, 43–57 10.1007/s13353-017-0424-3 29344903 PMC5799321

[325] Silva Ramos, E., Motori, E., Bruser, C., Kuhl, I., Yeroslaviz, A., Ruzzenente, B. et al. (2019) Mitochondrial fusion is required for regulation of mitochondrial DNA replication. PLoS Genet. 15, e1008085 10.1371/journal.pgen.1008085 31170154 PMC6553695

[326] Fratter, C., Gorman, G.S., Stewart, J.D., Buddles, M., Smith, C., Evans, J. et al. (2010) The clinical, histochemical, and molecular spectrum of PEO1 (Twinkle)-linked adPEO. Neurology 74, 1619–1626 10.1212/WNL.0b013e3181df099f 20479361 PMC2875130

[327] Remtulla, S., Emilie Nguyen, C.T., Prasad, C. and Campbell, C. (2019) Twinkle-associated mitochondrial DNA depletion. Pediatr. Neurol. 90, 61–65 10.1016/j.pediatrneurol.2018.08.007 30391088

[328] Koskinen, T., Sainio, K., Rapola, J., Pihko, H. and Paetau, A. (1994) Sensory neuropathy in infantile onset spinocerebellar ataxia (IOSCA). Muscle Nerve 17, 509–515 10.1002/mus.880170507 8159181

[329] Lonnqvist, T., Paetau, A., Valanne, L. and Pihko, H. (2009) Recessive twinkle mutations cause severe epileptic encephalopathy. Brain 132, 1553–1562 10.1093/brain/awp045 19304794

[330] Wang, H., Han, Y., Li, S., Chen, Y., Chen, Y., Wang, J. et al. (2021) Mitochondrial DNA depletion syndrome and its associated cardiac disease. Front. Cardiovasc. Med. 8, 808115 10.3389/fcvm.2021.808115 35237671 PMC8882844

[331] Durham, S.E., Brown, D.T., Turnbull, D.M. and Chinnery, P.F. (2006) Progressive depletion of mtDNA in mitochondrial myopathy. Neurology 67, 502–504 10.1212/01.wnl.0000227961.55640.2f 16894115

[332] Mathews, C.K. and Song, S. (2007) Maintaining precursor pools for mitochondrial DNA replication. FASEB J. 21, 2294–2303 10.1096/fj.06-7977rev 17403938

[333] Alonzo, J.R., Venkataraman, C., Field, M.S. and Stover, P.J. (2018) The mitochondrial inner membrane protein MPV17 prevents uracil accumulation in mitochondrial DNA. J. Biol. Chem. 293, 20285–20294 10.1074/jbc.RA118.004788 30385507 PMC6311524

[334] Sebastian, D., Palacin, M. and Zorzano, A. (2017) Mitochondrial dynamics: coupling mitochondrial fitness with healthy aging. Trends Mol. Med. 23, 201–215 10.1016/j.molmed.2017.01.003 28188102

[335] Hudson, G., Amati-Bonneau, P., Blakely, E.L., Stewart, J.D., He, L., Schaefer, A.M. et al. (2008) Mutation of OPA1 causes dominant optic atrophy with external ophthalmoplegia, ataxia, deafness and multiple mitochondrial DNA deletions: a novel disorder of mtDNA maintenance. Brain 131, 329–337 10.1093/brain/awm272 18065439

[336] Dölle, C., Flones, I., Nido, G.S., Miletic, H., Osuagwu, N., Kristoffersen, S. et al. (2016) Defective mitochondrial DNA homeostasis in the substantia nigra in Parkinson disease. Nat. Commun. 7, 13548 10.1038/ncomms13548 27874000 PMC5121427

[337] Wallace, D.C. (1989) Mitochondrial-DNA mutations and neuromuscular disease. Trends Genet. 5, 9–13 10.1016/0168-9525(89)90005-X 2652392

[338] deGrey, A.D.N.J. (1997) A proposed refinement of the mitochondrial free radical theory of aging. Bioessays 19, 161–166 10.1002/bies.950190211 9046246

[339] Diaz, F., Bayona-Bafaluy, M.P., Rana, M., Mora, M., Hao, H. and Moraes, C.T. (2002) Human mitochondrial DNA with large deletions repopulates organelles faster than full-length genomes under relaxed copy number control. Nucleic Acids Res. 30, 4626–4633 10.1093/nar/gkf602 12409452 PMC135822

[340] Gitschlag, B.L., Kirby, C.S., Samuels, D.C., Gangula, R.D., Mallal, S.A. and Patel, M.R. (2016) Homeostatic responses regulate selfish mitochondrial genome dynamics in C. elegans. Cell Metab. 24, 91–103 10.1016/j.cmet.2016.06.008 27411011 PMC5287496

[341] Baines, H.L., Stewart, J.B., Stamp, C., Zupanic, A., Kirkwood, T.B.L., Larsson, N.G. et al. (2014) Similar patterns of clonally expanded somatic mtDNA mutations in the colon of heterozygous mtDNA mutator mice and ageing humans. Mech. Ageing Dev. 139, 22–30 10.1016/j.mad.2014.06.003 24915468 PMC4141908

[342] Elson, J.L., Samuels, D.C., Turnbull, D.M. and Chinnery, P.F. (2001) Random intracellular drift explains the clonal expansion of mitochondrial DNA mutations with age. Am. J. Hum. Genet. 68, 802–806 10.1086/318801 11179029 PMC1274494

[343] Kowald, A. and Kirkwood, T.B.L. (2014) Transcription could be the key to the selection advantage of mitochondrial deletion mutants in aging. Proc. Natl Acad. Sci. U.S.A. 111, 2972–2977 10.1073/pnas.1314970111 24569805 PMC3939916

[344] Kowald, A. and Kirkwood, T.B.L. (2018) Resolving the enigma of the clonal expansion of mtDNA deletions. Genes 9, 126 10.3390/genes9030126 29495484 PMC5867847

[345] Insalata, F., Hoitzing, H., Aryaman, J. and Jones, N.S. (2022) Stochastic survival of the densest and mitochondrial DNA clonal expansion in aging. Proc. Natl Acad. Sci. U.S.A. 119, e2122073119 10.1073/pnas.2122073119 36442091 PMC9894218

[346] Greaves, L.C., Nooteboom, M., Elson, J.L., Tuppen, H.A.L., Taylor, G.A., Commane, D.M. et al. (2014) Clonal expansion of early to mid-life mitochondrial DNA point mutations drives mitochondrial dysfunction during human ageing. PLoS Genet. 10, e1004620 10.1371/journal.pgen.1004620 25232829 PMC4169240

[347] Tuppen, H.A.L., Reeve, A.K. and Vincent, A.E. (2023) Single cell analysis of mitochondrial DNA deletions. Methods Mol. Biol. 2615, 443–463 10.1007/978-1-0716-2922-2_29 36807808

[348] Bender, A., Krishnan, K.J., Morris, C.M., Taylor, G.A., Reeve, A.K., Perry, R.H. et al. (2006) High levels of mitochondrial DNA deletions in substantia nigra neurons in aging and Parkinson disease. Nat. Genet. 38, 515–517 10.1038/ng1769 16604074

